# Mechanisms of copper metabolism and cuproptosis: implications for liver diseases

**DOI:** 10.3389/fimmu.2025.1633711

**Published:** 2025-07-30

**Authors:** Haoran Chen, Dongxuan Li, Huimin Zhang, Meiqi Zhang, Yumeng Lin, Haibei He, Aijun Liu, Shiming Shen, Yi Wang, Zhongyu Han

**Affiliations:** ^1^ Department of General Surgery, Chengdu Xinhua Hospital Affiliated to North Sichuan Medical College, Chengdu, China; ^2^ School of Basic Medical Sciences, Anhui Medical College, Anhui, China; ^3^ Nanjing Tongren Hospital, School of Medicine, Southeast University, Nanjing, China; ^4^ Zhongda Hospital, Southeast University, Nanjing, China

**Keywords:** copper metabolism, cuproptosis, Wilson disease, alcoholic liver disease, non-alcoholic fatty liver disease, acute liver injury, hepatocellular carcinoma

## Abstract

Copper is an essential trace element in the human body, involved in various biological processes, including cell metabolism, nerve development, and immune function. Its homeostasis is vital for maintaining normal cellular functions, and disruptions in copper homeostasis can lead to a wide range of diseases. Cuproptosis is a copper ion–dependent form of programmed cell death that leads to abnormal oligomerization of lipoylated proteins and dysfunction of iron-sulfur cluster proteins in the mitochondrial tricarboxylic acid (TCA) cycle, thereby triggering intracellular oxidative stress and proteotoxic stress. In this review, we have delved into the mechanisms of copper metabolism and cuproptosis, as well as their roles in several liver diseases, including Wilson disease (WD), alcoholic liver disease (ALD), non-alcoholic fatty liver disease (NAFLD), acute liver injury (ALI), and hepatocellular carcinoma (HCC), as well as their therapeutic potential.

## 1 Introduction

Copper is an essential trace element in the human body. Although its concentration in the body is relatively low, it plays a crucial role in many important biological functions, such as cell metabolism, nerve development, and immune function (1). In recent years, the crucial role of copper homeostasis in human health has increasingly drawn attention. The concentration of copper ions within cells must be maintained at a precise homeostatic level, which is vital for normal cellular physiological functions. Under physiological conditions, copper ion absorption, distribution, storage, and excretion are strictly controlled. However, if copper homeostasis is disturbed, cells may experience a cascade of metabolic disorders, leading to various diseases.

Programmed cell death (PCD) is an essential process for maintaining tissue stability and developmental equilibrium. Under normal physiological cycles or various pathological conditions, organisms initiate different mechanisms of PCD to maintain homeostasis. The abnormal regulation of these mechanisms can trigger the development of various diseases. To date, several PCDs have been identified, such as apoptosis, necroptosis and ferroptosis, etc (2). Each type of PCD follows its unique mechanisms, contributing to the regulation of cell fate, and thus playing crucial roles in maintaining tissue homeostasis, immune responses, and disease progression.

In 2022, Tsvetkov et al. reported that elesclomol, a CI, could trigger a novel copper-dependent PCD, leading to the demise of ABC1 cells (3). This type of PCD is unique and cannot be reversed by inhibiting caspase-3 (apoptosis), necroptosis-1 (necroptosis), or ferrostatin-1 (ferroptosis). Consequently, this new mechanism of PCD has been termed cuproptosis. Cells undergoing cuproptosis display a spectrum of unique morphological alterations characterized by the shrinkage of mitochondria, reduction of the inner mitochondrial membrane, and the formation of large vacuoles; the endoplasmic reticulum becomes loosely structured and disordered; and the cell membrane ruptures (4). The excessive accumulation of copper within cells severely disrupts a series of copper-dependent metabolic processes, including key stages in the tricarboxylic acid (TCA) cycle and mitochondrial electron transfer chain (mtETC) (5). The aberrant progression of these metabolic activities ultimately disrupts the cell membrane’s integrity, causing the leakage of intracellular substances and resulting in cuproptosis. Cuproptosis plays a crucial role in various diseases, such as cancers, cardiovascular diseases, and pulmonary diseases (6–8). Moreover, as an immunogenic form of PCD, modulating cuproptotic regulators and cuproptosis is one of the key strategies to regulate immune cell infiltration and impact the prognosis of cancer patients (9–11).

Given the critical role of the liver in copper metabolism, research on copper homeostasis in various liver diseases has advanced significantly in recent years (12). However, the underlying mechanisms still require further exploration by scientific researchers to be fully elucidated. In this manuscript, we focus on the mechanisms of copper metabolism and cuproptosis, and explore their roles in various liver diseases, including Wilson disease (WD), alcoholic liver disease (ALD), non-alcoholic fatty liver disease (NAFLD), acute liver injury (ALI), and hepatocellular carcinoma (HCC).

## 2 Copper metabolism

Copper is a vital trace element for the human body, typically acquired through diet. These food sources supply the body with the required copper levels to support essential functions, such as angiogenesis, neuroregulation, and energy metabolism, etc (1). Copper is primarily obtained from food as copper ions (Cu^+^ and Cu^2+^), and is absorbed by intestinal epithelial cells (IECs) (13). Here, Cu^2+^ is reduced by six transmembrane epithelial antigen of the prostate (STEAP) and duodenal cytochrome b (DCYTB), and subsequently, the absorption of Cu^+^ is facilitated by copper transport protein 1 (CTR1), which is situated on the surface of IECs (14–16). The absorption of copper is regulated by multiple factors. IECs secrete the intestinal mucus layer, which serves as a physical barrier for the small intestine and also regulates the absorption of nutrients (17). Mucins within the mucus layer are important regulators of copper absorption, modulating the solubility and diffusion rate of copper ions via their copper-binding sites (18). The trace element zinc also regulates copper absorption. Zinc can bind to mucins, and exhibiting a higher binding affinity than copper (19). Excessive zinc can competitively inhibit copper absorption. In addition, recent research has indicated that zinc transporter 1 (ZnT1) has the capability to directly transport Cu^2+^ (20). Conditional knockout of ZnT1 in IECs can lead to a decrease in intracellular Cu^2+^ levels.

Copper absorbed by IECs can serve as a cofactor for enzymes to modulate the intracellular redox state, thereby sustaining the cells’ functionality and homeostasis. Additionally, it can be transported out of the cells via the ATP7A transporter (21). Copper in the bloodstream is transported by binding to companion proteins and delivered to target cells throughout the body (22). Similar to IECs, the process of copper entering target cells is also mediated by CTR1.

**Figure 1:**
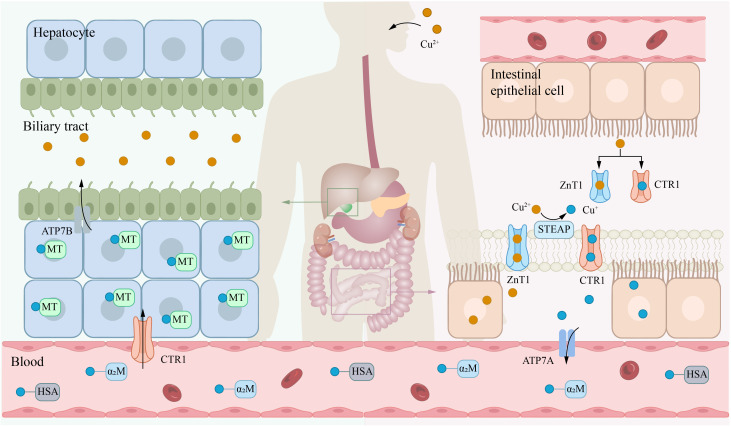
Systemic metabolism of copper. Copper is primarily absorbed in the small intestine, transported via the bloodstream to the liver for storage, and ultimately excreted into bile. The transporter CTR1 mainly facilitates the uptake of copper(I) (Cu^+^), while ZnT1 can transport copper(II) (Cu^2+^). Notably, zinc(II) ions (Zn^2+^) and Cu^2+^ compete for binding to ZnT1. In the blood, Cu^+^ is predominantly bound to HSA and α_2_M. Within the biliary system, copper ions are excreted primarily via the ATP7B. α_2_M, α_2_-macroglobulin; ATP7A, ATPase copper transporter 7A; CTR1, copper transport protein 1; HAS, human serum albumin; STEAP, six-transmembrane epithelial antigen of the prostate.

The liver is essential for copper metabolism. Copper that enters the bloodstream travels to the liver via the portal vein and subsequently enters hepatocytes through CTR1. In the liver, copper is incorporated into ceruloplasmin (CP), which is secreted by the liver and serves as the primary copper carrier protein. CP has a relatively short half-life, with fully copper-loaded CP (holo-CP) having a half-life of 4–5 days, while copper-free CP (apo-CP) has a half-life of approximately 5 hours. This dynamic regulation allows CP levels to fluctuate in response to changes in the hepatic copper pool, thereby maintaining systemic copper homeostasis. ATP7B is a transmembrane copper-transporting ATPase primarily expressed in the liver and is vital for maintaining overall copper balance in the body (23). ATP7B transports cytosolic copper ions to the Golgi apparatus, where they are loaded onto the precursor of CP to form functional CP, which is then secreted into the bloodstream (24). Additionally, when hepatic copper levels are elevated, ATP7B facilitates secrete excess copper into the bile for excretion, thereby maintaining copper homeostasis (**Figure 1**) (25).

**Figure 2:**
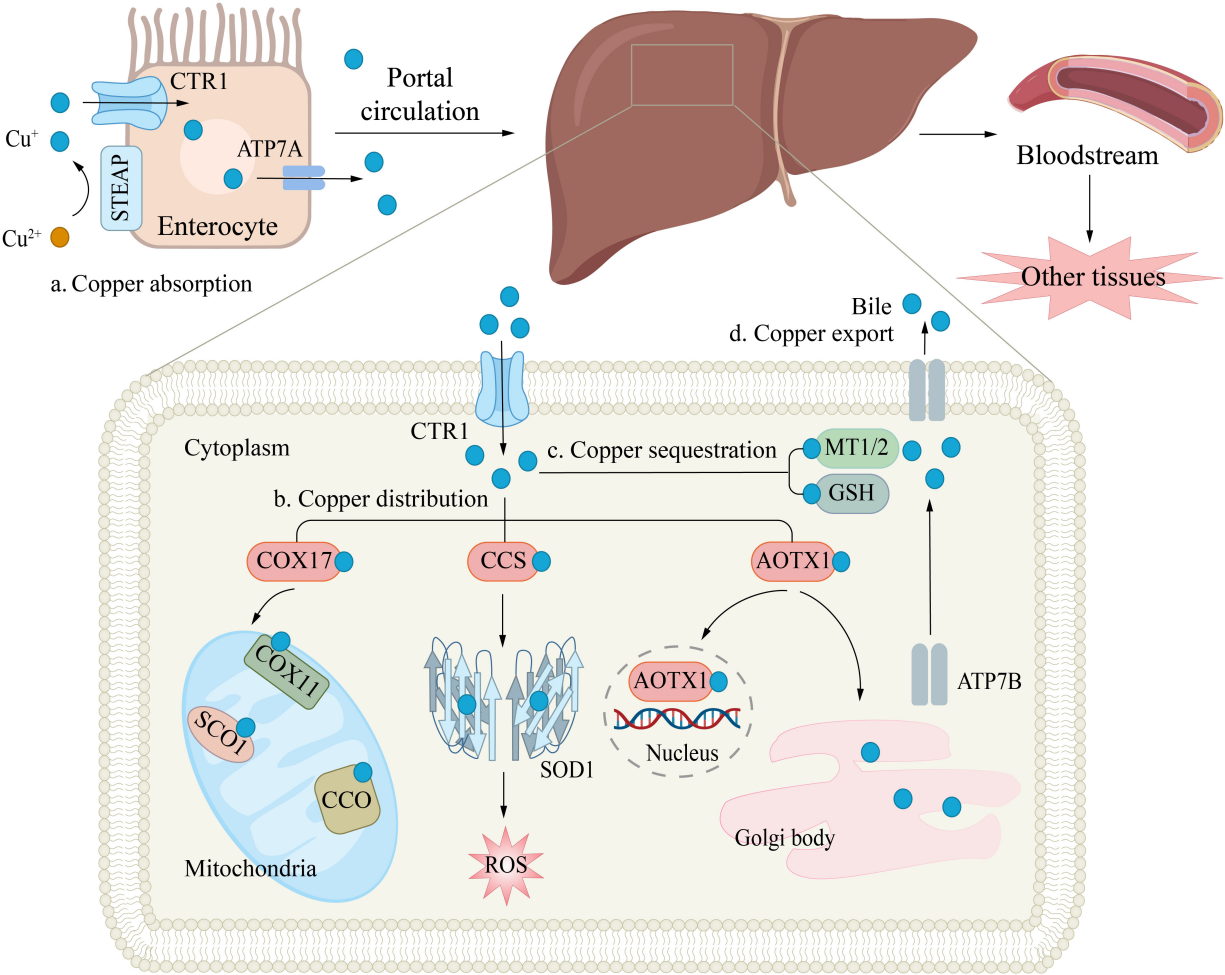
Copper Transport and Cellular Functions. **(a)** Copper absorption. Copper uptake in enterocytes is mediated by the CTR1 transporter, while ATPase copper transporter 7A (ATP7A) facilitates its efflux into the portal circulation. **(b)** Copper distribution. In hepatocytes, copper plays a critical role in antioxidant defense (e.g., by binding to superoxide dismutase, SOD) and supports mitochondrial energy production. The copper chaperones CCS and cytochrome c oxidase copper chaperone 17 (COX17) deliver Cu^+^ to specific targets: CCS directs copper to cytosolic SOD1, enhancing free radical scavenging; COX17 transports copper to mitochondrial COX, aiding ATP synthesis. Intracellular copper shuttling is regulated by ATOX1, which distributes copper to: The nucleus; ATPB in the trans-Golgi network. **(c)** Copper sequestration. Excess copper binds to MT1/2 or GSH and is stored in the cytosol to prevent oxidative damage. **(d)** Copper export. Finally, copper enters the bloodstream and is delivered to other tissues and organs.

In the bloodstream, copper ions transported to target cells by carrier proteins such as albumin and CP. At the surface of target cells, Cu²^+^ is reduced by STEAP and DCYTB (14, 15), and subsequently transported into the cell via CTR1 (26). Upon entering the cells, the storage, distribution, and efflux of copper ions are tightly regulated by various mechanisms. Metallothionein 1/2 (MT-1/2), which are rich in cysteine, can bind copper ions through their thiol groups (-SH), thereby mediating the storage of copper within cells (27). Moreover, copper ions can also be transported to specific target proteins via distinct chaperone proteins to exert their biological functions. For example, cytochrome c oxidase 17 (COX17) can transport copper to cytochrome c oxidase (CCO), participating in mitochondrial function and oxidative phosphorylation (28); copper chaperone for superoxide dismutase (CCS) can deliver copper ions to superoxide dismutase 1 (SOD1), thereby enhancing its antioxidant activity against reactive oxygen species (ROS) (29); antioxidant 1 copper chaperone (ATOX1) can shuttle copper to ATP7A or ATP7B, thereby aiding in the synthesis of CP and other copper-requiring enzymes, or promoting copper efflux to preserve intracellular copper homeostasis (**Figure 2**) (30–32). These processes collectively form an intricate regulatory network of intracellular copper metabolism, ensuring the rational distribution and utilization of copper ions, thereby maintaining normal cellular physiological functions.

## 3 Cuproptosis

Intracellular copper ion levels must be meticulously controlled to remain within a precise homeostatic range (33). When these levels surpass the physiological threshold, copper ions can exert toxic effects. Over four decades ago, biologists found that excess copper could cause cell death (34). Patients with WD stand at the forefront of copper biology and have provided key insights into cell death (35). They accumulate excess copper, leading to extensive hepatocyte death (36). The link between copper dysregulation and cell death is crucial in rare diseases like WD and also has significant implications for more common conditions, including cancer. However, the exact molecular mechanisms remain to be fully understood. Copper ionophores (CIs) are lipophilic molecules capable of reversibly binding to copper. They can traverse the plasma and mitochondrial membranes, delivering copper ions to specific intracellular locations (37). CIs are widely used in the study of intracellular copper transport and the elucidation of related mechanisms. The most commonly used CIs are Disulfiram (DSF) and Elesclomol (ES). DSF was initially employed for alcohol abstinence therapy but was later discovered to function as a CI, inducing copper-dependent antitumor effects (38). ES, originally developed by Synta Pharmaceuticals, was initially believed to induce tumor cell apoptosis through oxidative stress (39). Subsequent research revealed that ES serves as a CI, transporting Cu^2+^ into the mitochondria. The reduction of Cu^2+^ within the mitochondria enhances the production of ROS, thereby contributing to its antitumor mechanisms (40). With the in-depth research on CIs, in 2019, Tsvetkov et al. reported that by replacing glucose with galactose to alter cellular metabolism and increase mitochondrial respiration (Hi-mito state), tumor cells could acquire resistance to proteasome inhibitors. Intriguingly, in this metabolic state, cells become more sensitive to ES (41, 42). Further analysis revealed that the mitochondrial reductase ferredoxin 1 (FDX1) is a direct target of ES, leading to a unique form of copper-dependent PCD (41, 42). Subsequently, in 2022, they further elucidated that this PCD results from copper binding to lipoylated components within the mitochondria. Specifically, within the cell, particularly in the mitochondria, excess copper leads to abnormal aggregation of lipoylated proteins, disrupts iron-sulfur (Fe-S) cluster proteins involved in mitochondrial respiration, triggers proteotoxic stress responses, and results in cell death (3). Notably, this cell death pathway is resistant to inhibition by established PCDs inhibitors, and it has been termed cuproptosis (3).

Mitochondria are a critical target for cuproptosis. Tsvetkov et al. reported that cells highly dependent on mitochondrial respiration exhibit significantly greater sensitivity to CIs than those primarily relying on glycolysis (3). In addition, mitochondrial antioxidants and mitochondrial function inhibitors can both reduce this sensitivity. Furthermore, inhibiting key enzymes in cellular aerobic glycolysis, such as glyceraldehyde-3-phosphate dehydrogenase (GAPDH), can also increase the sensitivity to cuproptosis (43). Cuproptosis cells exhibit morphological features similar to apoptosis cells, including mitochondrial atrophy, plasma membrane rupture, and chromatin disruption (44). However, the underlying mechanisms are entirely different. Mechanistically, cuproptosis is initiated by the oligomerization of lipoylated components within the mitochondrial, rather than through the activation of caspase proteins.

Lipoylation is a conserved post-translational modification that regulates protein function by covalently attaching lipoic acid to lysine residues (45). To date, all known lipoylated proteins are key metabolic enzymes involved in the TCA cycle, such as dihydrolipoyl transacetylase (DLAT), dihydrolipoyllysine-residue succinyltransferase (DLST), pyruvate dehydrogenase complex component X (PDHX), and glycine cleavage system protein H (GCSH) (45). The lipoylated proteins are indispensable for the enzymatic activities of the energy metabolism complexes. However, the lipoic acid molecule contains two thiol groups (-SH), so proteins modified by lipoylation will also carry thiol groups, which endows them with a high affinity for copper ions (46, 47). Copper ions binding to thiol groups can induce thiol-dependent oligomerization of lipoylated proteins, leading to proteotoxic stress and culminating in cuproptosis (3, 48).

The primary regulators of protein lipoylation are lipoyltransferase 1/2 (LIPT1/2) and lipoic acid synthase (LIAS). LIPT2 can transfer the octanoyl group from acyl-carrier protein to GCSH, which serves as a precursor for lipoic acid synthesis (49). LIAS, which contains an Fe-S cluster, is a crucial enzyme in lipoic acid synthesis that inserts two sulfur atoms into carbons 6 and 8 of the octanoyl group to form complete lipoic acid (50). Subsequently, LIPT1 transfers lipoic acid from GCSH to the lysine residues of target proteins, completing the lipoylation modification (47, 51). In current studies, DLAT, LIPT1 and LIAS are implicated in positively regulating cuproptosis, with their expression levels being closely related to cuproptosis activity (3, 52–54). In contrast, the direct role of LIPT2 in cuproptosis is still unclear. However, given its essential function in lipoylation, LIPT2 may exert an indirect influence on cuproptosis by modulating the lipoylation pathway.

**Figure 3:**
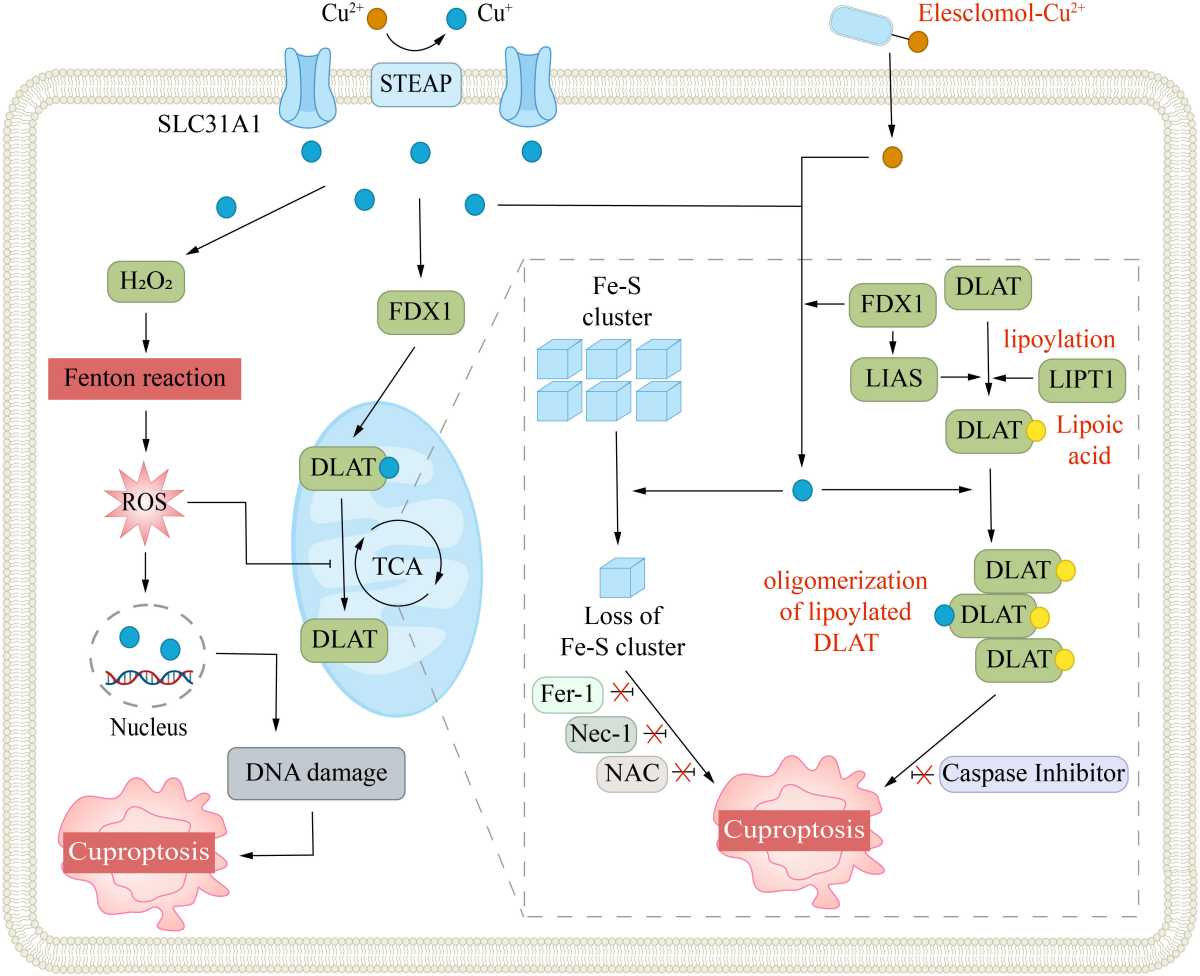
Mechanism of cuproptosis. Cuproptosis is triggered by excessive intracellular copper accumulation, which occurs either via SLC31A1-mediated uptake or through copper ionophores. Elevated copper levels promote ROS generation via the Fenton reaction, leading to DNA damage. Copper ionophores (e.g., elesclomol) facilitate copper transport into cells, where it binds to lipoylated enzymes in the mitochondrial TCA cycle, such as DLAT. This interaction causes protein aggregation and disrupts iron-sulfur cluster stability. The FDX1/LIAS pathway, which regulates protein lipoylation, further contributes to mitochondrial dysfunction. Collectively, these disruptions induce proteotoxic stress, culminating in cell death. Inhibitors targeting ferroptosis (Fer-1), necroptosis (Nec-1), or oxidative stress (NAC) do not prevent this form of cell death.

FDX1 is another pivotal regulator of cuproptosis. FDX1 is a small protein from the ferredoxin family and contains an Fe-S cluster (55). It serves as an electron donor and participates in the biosynthesis of steroids, heme, and Fe-S clusters. FDX1 can interact with ES-Cu to facilitate the reduction of Cu^2+^ and release Cu^+^ into the mitochondrial matrix (41). This reduction reaction significantly amplifies the cytotoxicity of copper ions. Notably, copper overload can suppress FDX1 activity, leading to a decrease in the synthesis of Fe-S cluster proteins. Moreover, Cu^+^ can directly bind to Fe-S clusters, which in turn disrupt their synthesis and function (3). In addition, FDX1 is an upstream regulator in the lipoylation pathway. Dreishpoon et al. reported that FDX1 can directly interact with LIAS, acting as an electron donor to facilitate the synthesis of lipoic acid, rather than indirectly regulating it through modulation of Fe-S clusters (**Figure 3**) (56). The loss of FDX1 function, by affecting protein lipoylation, leads to the suppression of mitochondrial respiration, particularly under low-glucose conditions.

## 4 Abnormal copper metabolism and liver disease

The liver plays a central role in copper metabolism, including absorption, storage, transportation and excretion. Disruptions in these processes are linked to various liver diseases and can greatly affect immune responses and inflammation within the liver (57). Copper homeostasis finely regulates liver immunity. It is essential for immune cell function and host defense, but its imbalance can cause excessive immune activation and inflammation. Copper deficiency weakens the immune response to infections and damaged cell clearance, worsening liver injury (58–60). Conversely, excess copper can overactivate immune cells like Kupffer cells, T cells, and B cells, enhancing inflammation (7, 61). Cuproptosis, as an immunogenic PCD, leads to the release of damage-associated molecular patterns (DAMPs), promotes inflammation and the infiltration of immune cells, and aggravates hepatocyte damage (12, 62, 63). In addition, immune system abnormalities can disrupt copper metabolism by altering copper transporter and binding protein expression and activity (64). The complex interplay between copper metabolism and the immune system highlights their importance in liver diseases. Overall, damage to one or more stages of copper metabolism in the liver can lead to copper metabolism disorders, which in turn cause abnormal liver immunity and aggravate hepatocyte damage. In the following sections, we will discuss the role of abnormal copper metabolism in WD, ALD, NAFLD, ALI and HCC.

### 4.1 Abnormal copper metabolism in Wilson disease

Menkes disease (MD) and WD are genetic disorders associated with copper metabolism, characterized by mutations in the *ATP7A* or *ATP7B* genes, respectively. MD arises from *ATP7A* gene mutations, which leads to a reduction in functional ATP7A in IECs and severe systemic copper deficiency (65). The main symptoms of MD include neurological deterioration and growth retardation, with relatively mild liver symptoms. Copper deficiency also leads to reduced synthesis of copper-dependent enzymes like CP. In contrast, WD is caused by *ATP7B* gene mutations. Since ATP7B is involved in CP synthesis and copper excretion, its missense mutations often result in copper accumulation within the liver, causing severe hepatotoxicity and systemic symptoms (66). In WD patients, the hepatic copper concentration can be more than 10 times the normal level (67). Meanwhile, the reduction of holo-CP in the circulation leads to a shortened half-life of CP, resulting in not only excessive copper but also excessive iron in the liver of WD patients, which in turn causes iron-induced Fenton reactions and iron-related lipid peroxidation (68). Compared with copper, the presence of a certain level of CP means that iron accumulation is not a primary feature of WD.

In WD patients, the absence of ATP7B expression in IECs leaves copper absorption unaffected. Additionally, the transfer of copper from IECs to the liver is also not influenced by *ATP7B* mutations (69). This allows copper to easily accumulate in large amounts in the livers, subsequently triggering a series of pathological changes. Mitochondrial alterations often manifest in the early stages, such as the separation and expansion of the mitochondrial membrane, deformation, swelling, and the appearance of vacuoles in mitochondrial (70). Similar structural changes can also be observed in *ATP7B* ^-/-^ rats (71, 72). As the disease advances, defects in the mtETC become increasingly pronounced, particularly in complex IV (73, 74). This leads to a diminished capacity for ATP production, thereby compromising cellular energy metabolism. Conversely, the Fenton reactions and ROS damage associated with copper and iron overload tend to occur at a later stage. The change in the glutathione (GSH)/glutathione disulfide (GSSG) ratio occurs at a later stage, reflecting a decline in antioxidant capacity (75).

Restricting copper intake in the diet can be somewhat helpful for patients with WD, but the current evidence is not sufficient to support a treatment plan based on dietary restrictions (76). At present, the treatment of WD mainly relies on copper chelators (CCs) and zinc salts (77). CCs can specifically bind to copper ions to form stable complex, which promotes the excretion of copper (78). D-penicillamine and trientine are first-line drugs for WD. D-penicillamine is the earliest used CC. It is an amino acid containing a thiol group, which can remove copper bound to albumin in the circulation and excrete it through urine (78). The side effects of D-penicillamine mainly include nephrotoxicity and bone marrow suppression, etc (79). Trientine is an alternative for patients who cannot tolerate D-penicillamine (80, 81). Another CC, tetrathiomolybdate (TTM), has also demonstrated potential in treating WD. In a phase II clinical trial, bis-choline TTM was found to rapidly reduce the levels of non-CP-bound copper in patients, and improve neurological symptoms and liver function (82). Zinc salts are another first-line treatment option for WD. As mentioned, zinc can competitively inhibit the absorption of copper (19). Li et al. reported that ZnT1 is involved in transporting both zinc and copper and is essential for zinc’s ability to inhibit copper absorption. ZnT1 has a unique inter-subunit disulfide bond that aids in the transport of Cu^2+^, and Zn^2+^ and Cu^2+^ share a major binding site on ZnT1 (83). Therefore, ZnT1 may be a potential target for zinc-based therapeutic approaches in treating WD.

### 4.2 Abnormal copper metabolism in alcohol liver disease

The liver is the core organ for ethanol metabolism. As a result, long-term alcohol consumption can easily lead to liver damage, a prevalent form of hepatic injury. Copper is an important fungicide in organic viticulture and also an important catalyst in the wine-making process (84). Copper significantly contributes to the progression of ALD. Lin et al. reported that dietary copper can improve the intestinal barrier integrity and hepatic injury in ALD model mice (85). Dietary copper supplementation can increase the expression of hypoxia-inducible factor-1α (HIF-1α) in the small intestine, thereby enhancing intestinal barrier stability through the upregulation of P-glycoprotein and tight junction proteins (86). Additionally, copper supplementation can also increase the levels of GPX1 and occludin, ameliorate oxidative stress, and mitigate ethanol-induced damage to the small intestine (85). Conversely, some studies have reported that copper can increase the liver damage caused by alcohol (87, 88). Monooxygenase DBH-like 1 (MOXD1) is closely related to the transport of copper into cells (89). A bioinformatics analysis has revealed that MOXD1 is a key gene in ALD, and inhibiting MOXD1 can improve inflammation in ALD mice (90). Hou et al. reported that the livers of ALD model mice exhibited significant infiltration of M1 macrophages, a notable decline in FDX1 expression, and a pronounced upsurge in CTR1 expression. They also pinpointed five potential biomarkers associated with M1 macrophages, ferroptosis, and cuproptosis in patients with alcoholic hepatitis: LUM, ALDOA, THBS2, COL3A1, and TIMP1 (91). In another study, bioinformatics analysis revealed that three cuproptosis-related genes (CRGs)—*DLAT*, *GLS*, and *CDKN2A*—are closely associated with ALD (92). Their expression were markedly elevated in both ALD patients and ALD model mice. *GLS* and *CDKN2A* exhibit a correlation with the p53 pathway, which can increase cellular sensitivity to cuproptosis through multiple pathways, such as inhibiting glycolysis, enhancing mitochondrial function and Fe-S cluster biogenesis, and inhibiting the SLC7A11-glutathione peroxidase 4 (GPX4)-GSH pathway to reduce GSH levels (93–95). In addition, the CRGs are associated with the hepatic infiltration of macrophages and CD8^+^ T cells, and may activate the complement pathway, thereby amplifying intrahepatic inflammation (92). Overall, copper significantly impacts ALD pathogenesis via multiple pathways.

### 4.3 Abnormal copper metabolism in non-alcoholic fatty liver disease

NAFLD is a highly prevalent liver disease. Its core pathological feature is the excessive lipid accumulation within hepatocytes, which primarily results from overconsumption of dietary fats, excessive fatty acid influx, and elevated *de novo* lipogenesis (96). The long-term lipid accumulation leads to mitochondrial oxidative stress. If left uncontrolled, this chronic damage can progressively develop into non-alcoholic steatohepatitis (NASH), and further induce fibrosis and HCC (97). NAFLD is closely associated with metabolic abnormalities. Patients typically have obesity, insulin resistance, or metabolic dysfunction (98). As the prevalence of these metabolic-related diseases increases, the incidence of NAFLD has also risen significantly, with approximately 38% of individuals affected by NAFLD (99). To emphasize the importance of metabolic dysfunction, NAFLD is also termed metabolic dysfunction-associated fatty liver disease (MAFLD) (100). Dysregulated copper metabolism is strongly associated with NAFLD and may intensify its pathological progression (101).

Abnormal copper homeostasis, including both elevated and deficient copper, can cause liver injury and exacerbate NAFLD progression (102). A study reported that serum copper levels correlate positively with NAFLD prevalence and the progression of fibrosis, with overweight women under the age of 60 having a higher susceptibility (103). In contrast, another study reported that elevated blood copper levels significantly protect male NAFLD patients, and this protective effect increases with the severity of NAFLD. In women, however, this protective effect is only observed in mild liver disease (104). A meta-analysis revealed that hepatic copper levels in NAFLD patients are significantly reduced. However, there is no significant correlation between serum copper levels, CP levels, and NAFLD (105). These contradictory results are closely tied to the complex pathogenesis of NAFLD and are also closely associated with the multifaceted physiological functions of copper. Copper can influence the progression of NAFLD through various pathways, such as regulating lipid metabolism, iron metabolism, mitochondrial function, oxidative stress, and cuproptosis. Jiang et al. reported that oral copper oxide nanoparticles cause copper accumulation in the liver, resulting in disordered hepatocyte arrangement, lipid vacuolation, and hepatic fibrosis (106). Mechanistically, CuO NPs increase bile acid (BA) reabsorption, disrupting BA homeostasis. This leads to BA accumulation and induces NAFLD. The expression of the pregnane X receptor (PXR) in the liver was significantly upregulated, correlating with BA accumulation and promoting lipid synthesis (106, 107). Additionally, Liu et al. reported that copper can induce inflammatory responses in the mouse liver by activating the mitogen-activated protein kinase (MAPK) and nuclear factor-kappa B (NF-κB) pathways, leading to hepatic dysfunction (108). Copper deficiency can reduce the expression of ferroportin, thereby affecting hepatic iron metabolism, causing hepatic iron deposition and promoting liver fibrosis (109). Copper deficiency can also lead to increased ROS, thereby exacerbating the pathological progression of NAFLD (110). In addition, copper deficiency can affect the key enzyme in fructose metabolism, such as ketohexokinase, while high fructose intake can impair copper absorption, leading to copper deficiency (111). The copper-fructose interaction can exacerbate NAFLD through multiple pathways, including promoting hepatic iron overload, mitochondrial dysfunction, and abnormal gut function (112).

Copper also affects various metabolic disorders that contribute to the development of NAFLD/MAFLD. For example, copper and zinc compete for binding to ZnT1 and mucins, which means that elevated copper levels can suppress zinc absorption (19, 20). Zinc is an important cofactor for insulin synthesis and secretion, and zinc deficiency can exacerbate insulin resistance (113). In addition, high levels of copper can also promote oxidative stress through the Fenton reaction, damage pancreatic β cells, and thereby promote insulin resistance (114). Additionally, copper serves as a cofactor for enzymes involved in lipid metabolism such as semicarbazide-sensitive amine oxidase (SSAO) and amine oxidase copper-containing 3 (AOC3) (115, 116). Abnormal levels of copper can lead to dysregulation of lipid metabolism and increased fat accumulation, thereby heightening the risk of obesity (117).

At present, studies exploring cuproptosis in NAFLD remain relatively few, and its mechanisms remain to be elucidated. Xu et al. reported that overexpression of the *LIAS* gene in leptin receptor-deficient mice significantly enhances the mitochondrial antioxidant capacity in the liver, reduces the levels of inflammatory factors, and alleviates the progression of NAFLD/NASH (118). Zhao et al. identified that *FDX1* is closely associated with the progression from NASH to HCC. FDX1 expression is significantly increased in areas of hepatocyte steatosis but is markedly decreased in HCC. Targeting FDX1 may help prevent the malignant progression of NAFLD (119). However, another bioinformatics analysis did not identify *FDX1* as a CRG associated with NAFLD. In their study, FDX1 showed no significant difference, whereas *NFE2L2*, *DLD*, and *POLD1* were identified as key CRGs and were closely related to the immune microenvironment (120). This further highlights the dynamic changes of FDX1 in the NAFLD-NASH-HCC progression, but this still requires more research for validation. Another bioinformatics analysis showed that six CRGs changed significantly in NAFLD. *NFE2L2*, *LIAS*, and *ATP7B* were significantly downregulated, while *DLD*, *DLAT*, and *PDHB* were highly upregulated (121). In summary, copper ions regulate the progression of NAFLD through various pathways, and the abnormal changes in their levels may exacerbate the pathological process of NAFLD.

### 4.4 Abnormal copper metabolism in acute liver injury

ALI is the extensive death of hepatocytes and inflammation that occur within a short period of time, leading to acute liver impairment. Common causes of ALI include hepatic ischemia-reperfusion (HIR), drug overdose, excessive alcohol consumption, heavy metal poisoning, autoimmune hepatitis, and viral hepatitis (122). Studies have reported that excessive copper intake or exposure can directly cause ALI (88, 123, 124). Other factors can also cause abnormal copper levels in the liver, affecting mitochondrial function within hepatocytes and pathways like oxidative stress and cuproptosis, thereby exacerbating ALI.

HIR is often accompanied by various PCDs, including cuproptosis (125). Inhibitors of cuproptosis have been shown to improve liver injury, whereas inducers can exacerbate it. Bioinformatics analysis has identified some characteristic CRGs that are highly expressed in HIRI (126). Acetaminophen (APAP) is frequently used as an antipyretic and anti-inflammatory drug. However, APAP exhibits a relatively limited therapeutic window, with overdose being a critical contributor to ALI. Guo et al. reported that after APAP treatment, the copper content in the liver is significantly increased (127). Bioinformatics analysis identified four CRGs that are closely associated with acetaminophen-induced liver injury (AILI): *PDHA1*, *SDHB*, *NDUFB2*, and *NDUFB6*. Their expression is markedly reduced in AILI and is correlated with the infiltration of M1 macrophages (127). Luo et al. reported that in concanavalin A-induced immune-mediated ALI, Merestinib can directly bind to Nrf2, thereby reducing oxidative stress. Additionally, it regulates copper homeostasis in hepatocytes, inhibits cuproptosis, and alleviates liver damage (128).

Viral hepatitis can also cause acute liver injury. However, studies on cuproptosis in viral hepatitis are still limited at present. Nevertheless, research has shown that in patients with chronic hepatitis B, serum copper levels are significantly reduced, antioxidant capacity is weakened, and ROS levels are significantly increased (129, 130). In chronic hepatitis C patients, hepatic copper levels correlate positively with the extent of hepatic fibrosis, yet serum copper levels show no significant difference (131). These findings suggest that copper metabolism disorders might contribute to the pathogenesis of viral hepatitis and are worthy of further detailed research.

### 4.5 Abnormal copper metabolism in hepatocellular carcinoma

HCC is the most common type of primary liver cancer (132). The pathogenesis of HCC is highly complex, encompassing genetic factors, metabolic disorders, viral infections, and environmental exposures. These diverse risk factors contribute to liver cirrhosis development, fostering an environment conducive to the progression of HCC (133). Copper is vital for maintaining mitochondrial function and significantly impacts energy metabolism. Given that cancer cells proliferate rapidly, it is predictable that they require more copper than normal cells to satisfy their energy requirements. Moreover, since copper metabolism abnormalities play a vital role in various chronic liver diseases, the relevance of copper to HCC development is self-evident. Research has demonstrated that copper concentrations are markedly higher in various cancers (134–138). Li et al. reported that the expression levels of most copper metabolism-related pathways are decreased in HCC patients. The expression of MTs genes related to copper ion detoxification is significantly decreased in HCC (139).

In HCC, the abnormal elevation of copper ion levels can influence the biological behavior of tumors through various mechanisms (140). A bioinformatics analysis revealed that HCC patients presenting a high-copper phenotype typically exhibit higher pathological grades and poorer prognoses compared to those with a low-copper phenotype. Additionally, the high copper phenotype was characterized by higher expression of immune checkpoint genes, resulting in a poorer response to immunotherapy (141). Davis et al. reported that copper levels in HCC tumor tissues were significantly higher than those in adjacent liver tissues. The overexpression of CTR1 promoted the proliferative and migratory capacities of HCC cells. Conversely, silencing CTR1 or treatment with TTM significantly reduced glycolytic gene expression and downstream metabolite utilization in HCC cells, thus inhibiting cell survival in hypoxic conditions (142). Excess copper can promote the proliferation of HCC through the MYC-CTR1 axis. *MYC* is an important oncogene that can promote cell proliferation through the modulation of cell cycle-related gene expression (143). Dysregulation of MYC is crucial for the proliferation, invasion, and other processes of HCC (144). Porcu et al. reported that copper exposure upregulated MYC expression, and MYC could bind to the CTR1 promoter to promote its transcription. Silencing of CTR1 can offset the copper-induced cell proliferation (145). Excess copper within cells can also increase free radicals, exacerbate DNA damage, thereby promoting the proliferation of tumor cells (146). SOD1-/- mice exhibit a higher incidence of HCC.

Angiogenesis is a vital pathway for the progression, invasion, and metastasis of HCC. Copper significantly influences angiogenesis and contributes to the malignant progression of various tumors (147, 148). Copper metabolizing MURR1 domain 3 (COMMD3) is a crucial regulator of copper metabolism, and its expression levels are closely correlated with intracellular copper ion concentrations (31). The high expression of COMMD3 is associated with adverse outcomes in a range of tumors and is related to the tumor’s abilities of migration, invasion, and angiogenesis (149, 150). Zhu et al. reported that overexpression of COMMD3 significantly increased the expression of vascular endothelial growth factor (VEGF), p-VEGFR2/VEGFR2, HIF-1α, and NF-κB in HCC cells, thereby promoting tumor growth and angiogenesis (151). Conversely, downregulation of COMMD3 effectively inhibited this tumor-promoting process. Studies have reported that copper chelation therapy can improve the progression and angiogenesis of HCC (152–154). Copper can also bind to CD147, promoting its self-aggregation and thereby facilitating the proliferation, invasion, and metastasis of HCC (155). CD147 is overexpressed in various malignant cancers and can activate matrix metalloproteinases (MMPs) -2, -3, and -9, thereby facilitating the migration of HCC cells. Additionally, CD147 can regulate the proliferation of HCC cells through the phosphoinositide-3-kinase (PI3K)/protein kinase B (Akt) and p53 signaling pathway (155, 156).

Targeting copper homeostasis has become a potential therapeutic approach for treating HCC. By regulating the intracellular levels of copper ions or leveraging copper-induced PCD, the progression of HCC can be effectively inhibited. Numerous studies have elucidated diverse mechanisms to target copper ions for treating HCC. HCC cells need a higher level of copper ions to promote their proliferation, invasion, and metastatic ability. However, deviations from this optimal concentration—either a reduction or an increase—can effectively impede the progression of HCC.

CCs have been proven to significantly reduce the proliferation and invasive capacity of HCC cells by inhibiting the activity of copper ions (142, 152). Moreover, excess copper ions can inhibit the progression of HCC through various mechanisms. For example, an appropriate amount of copper can promote HCC through ROS-dependent mechanisms. However, an excess of copper can increase the intracellular ROS levels and induce apoptosis. Niu et al. reported that the copper (II) complex of salicylate phenanthroline [Cu(sal)(phen)] can significantly increase the ROS levels in HCC cells and promote apoptosis (157). The treatment with Cu(sal)(phen) significantly decreased Bcl-2 levels and triggered apoptosis in a dose-dependent manner. Similarly, Jiang et al. synthesized two Cu(II) 4-hydroxybenzoylhydrazone complexes, which can promote HCC apoptosis by increasing ROS generation and damaging mitochondrial DNA (158).

Cuproptosis is the primary pathway through which copper exerts its anticancer effects. Bioinformatics analyses have underscored the strong correlation of CRGs with the progression, prognosis, and tumor microenvironment (TME) of HCC (159–161). FDX1 can influence the prognosis of HCC by modulating cuproptosis and the TME (162). High expression of FDX1 promotes susceptibility to cuproptosis in HCC cells, enhances the infiltration of NK cells, Th1 cells, and macrophages, and increases sensitivity to oxaliplatin (163, 164). In contrast, low expression of FDX1 enhances tolerance to Cu^2+^ and correlates with a worse prognosis in HCC (162). In addition, Sun et al. reported that knockdown of *FDX1* promotes mitophagy, activates the PI3K/Akt pathway and facilitates HCC progression (165). DLAT also promotes the proliferation of HCC (166). Li et al. reported that maternal embryonic leucine zipper kinase (MELK) is highly expressed in HCC and promotes DLAT via the PI3K/Akt pathway, improving mitochondrial function (167). ES treatment inhibits MELK-induced HCC progression by promoting DLAT oligomerization and inducing cuproptosis. In addition, LIPT1, LIAS, and GLS can also promote the malignant progression of HCC (168–170).

Moreover, cuproptosis and ferroptosis are synergistic in HCC (171). Ferroptosis is an iron-dependent PCD, characterized by the elevation of intracellular iron ion levels that generate massive ROS through the Fenton reaction, along with the depletion of the antioxidant GSH, which subsequently trigger lipid peroxidation. This damages the cell and mitochondrial membranes, and ultimately causing cell death (172). Ren et al. reported that DSF-Cu can promote the iron ion levels and lipid peroxidation in HCC cells, thereby facilitating ferroptosis. DSF-Cu reduced the GSH levels and FDX1 expression, while simultaneously promoting ferroptosis and cuproptosis (173, 174). Combined treatment with the ferroptosis inducer sorafenib enhanced the cytotoxicity against HCC cells. In addition, sorafenib can also promote copper death induced by ES-Cu. Treatment with sorafenib can enhance the stability of FDX1 and reduce the level of GSH, and the enhanced effect can be reversed by treatment with GSH or TTM (175). Combining ferroptosis inducers with CIs could offer a new and potentially more effective approach to treating HCC.

**Figure 4:**
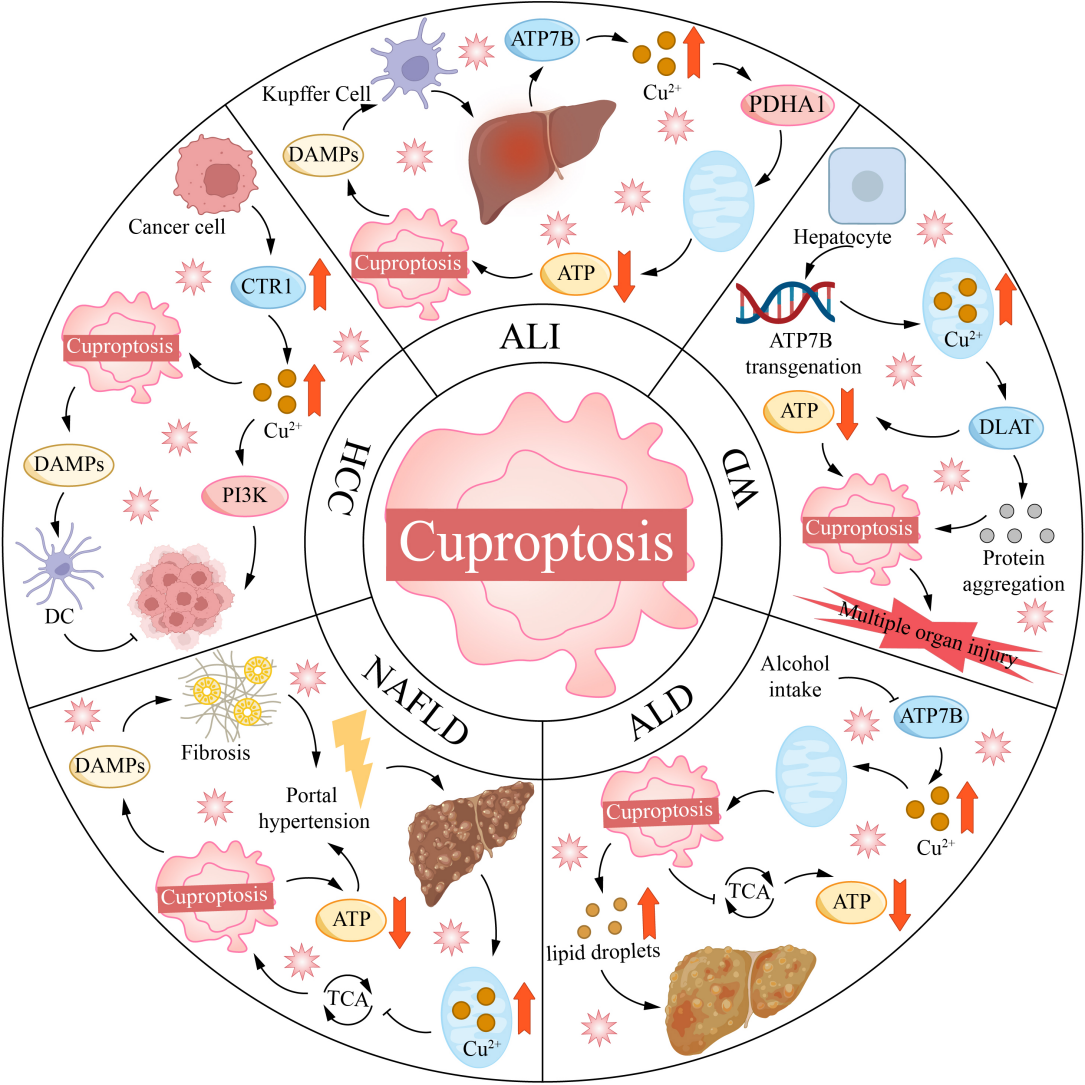
Copper mortality is associated with various liver diseases. ALI, acute liver injury; ALD, alcoholic liver disease; ATP7B, ATPase copper transporting 7B; CTR1, copper transporter 1; DAMPs, damage-associated molecular patterns; DC, dendritic cell; DLAT, dihydrolipoamide S-acetyltransferase; HCC, hepatocellular carcinoma; NAFLD, non-alcoholic fatty liver disease; PDHA1, [yruvate dehydrogenase E1 alpha 1 subunit; PI3K, phosphoinositide-3-kinase; TCA, tricarboxylic acid cycle; WD, Wilson disease.

In addition to directly promoting PCDs, copper can also modulate the immune microenvironment of HCC. Copper-triggered Cuproptosis and ferroptosis release DAMPs, thereby enhancing antitumor immunity. Mao et al. developed a prognostic model for HCC containing 9 CRGs. The low-risk group exhibited high expression of GLS, LIPT1, ATP7A, and ATP7B, as well as increased immune infiltration and sensitivity to immune checkpoint inhibitors (176). However, copper can also promote immunosuppression in the HCC TME. A study reported that DSF-Cu demonstrates antitumor effects in immunodeficient mice, but it promotes the upregulation of programmed death ligand-1 (PD-L1) in immunocompetent mice, leading to a less favorable therapeutic outcome (177). PD-L1 binds to PD-1 on T cells, delivering inhibitory signals to T cells, thereby suppressing their proliferation, activation, and cytokine secretion (178). DSF-Cu achieves this by inhibiting Poly (ADP-ribose) polymerase 1 (PARP1) activity, increasing the phosphorylation of GSK3β at the Ser9 site, thereby inhibiting GSK3β activity and reducing the degradation of PD-L1. However, when DSF/Cu is combined with anti-PD-1 antibodies, the expression of PARP1 and PD-L1 is decreased, while the expression of CD8^+^ T cells and granzyme B is increased, significantly enhancing antitumor efficacy (177). This offers a novel combinatorial therapeutic strategy for treating HCC (**Figure 4**).

## 5 Conclusion

Copper participates in numerous biological processes, and its balance is crucial for normal cellular functions. Imbalances in copper homeostasis can trigger a wide range of diseases. Cuproptosis, a copper ion-dependent PCD, and has been implicated in various diseases, where it can either exacerbate or mitigate disease progression, depending on the context. The liver is a key organ in copper metabolism, regulating its absorption, storage, transport, and excretion. In this manuscript, we focus on the mechanisms of copper metabolism and cuproptosis, and explore their significance in WD, ALD, NAFLD, ALI, and HCC.

Additionally, copper overload also activates cuproptosis, leading to mitochondrial dysfunction, activation of ferroptosis-related pathways, and the transmission of apoptotic signals, which further induce hepatocyte death. The extensive death of hepatocytes not only exacerbates liver damage but also triggers inflammatory responses, triggering the release of inflammatory mediators and the infiltration of immune cells. Chronic inflammatory stimulation, along with the repeated injury and regeneration of hepatocytes, leads to liver fibrosis, cirrhosis and drives the progression of HCC. Therefore, targeting the regulation of intracellular copper homeostasis is a key strategy for treating liver diseases.

Currently, the common copper-targeting drugs used in clinical practice mainly include CCs and CIs. CCs can bind to copper ions and facilitate their excretion. They are widely used to treat copper-related disorders like WD. CIs can transport copper ions across cell membranes, increase intracellular copper, and promote cuproptosis. This mechanism has potential applications in HCC treatment. However, there are still many gaps in the current research. CIs, such as DFS and ES, have demonstrated therapeutic potential in HCC, but most studies are still limited to animal or *in vitro* experiments, with a lack of relevant clinical research. Some copper-targeting drugs still encounter challenges in overcome resistance to immunotherapies, and do not achieve the expected therapeutic effects. Moreover, due to the complexity of copper’s actions, further clinical studies are necessary to investigate how to precisely regulate copper levels in specific liver diseases. In addition, cuproptosis, along with ferroptosis, apoptosis, and autophagy, constitutes a network regulating cell fate. For instance, abnormal copper metabolism can cause redox imbalance, and such redox fluctuations are key factors in influencing other PCD pathways like ferroptosis and disulfidptosis (179–181). Further studies are required to investigate their interplay in liver diseases. Finally, copper is vital for normal cellular function. Finding methods to selectively target copper metabolism in liver diseases while minimizing harm to healthy cells and organs remains a significant challenge for future research.

Based on the current challenges, future research should prioritize several key aspects. Specifically, leveraging advanced imaging technologies alongside multi-omics sequencing of particular circulating biomarkers in the blood allows for the identification of biomarkers signaling copper metabolic disruption. These biomarkers can serve as early diagnostic indicators and facilitate the monitoring of disease progression and therapeutic outcomes. Similarly, identifying novel therapeutic drugs that specifically target copper metabolism is crucial. The primary objective is to develop drugs with heightened selectivity and efficacy while minimizing off-target toxicity to healthy cells. Researchers should meticulously assess the risk-benefit profile of these drugs to ensure they selectively modulate copper levels in diseased tissues without compromising the normal physiological functions of copper in healthy cells. Moreover, investigating the synergistic effects of copper-targeting therapies with other therapeutic approaches represents a promising avenue. Combining copper-based strategies with immunotherapies or metabolic drugs could potentially enhance treatment efficacy and address the limitations of single-therapy resistance. Furthermore, personalized treatment plans should be developed based on the genetic makeup, disease subtype, and individual copper metabolism status. Patient-stratification approaches that account for these variables can help tailor therapies to improve treatment precision and efficacy. By integrating these research directions, the scientific community can make significant progress in understanding copper-related liver diseases and developing more effective therapies to combat them.

In summary, copper metabolism and cuproptosis play crucial roles in the pathogenesis and treatment of various liver diseases. Clarifying the mechanisms that disrupt copper homeostasis in liver diseases will help in the development of new therapeutic strategies.

## Glossary

AILI: acetaminophen-induced liver injury
Akt: protein kinase B
ALD: alcoholic liver disease
ALI: acute liver injury
APAP: acetaminophen
AOC3: amine oxidase copper-containing 3
ATOX1: antioxidant 1 copper chaperone
BA: bile acid
CC: copper chelator
CCO: cytochrome c oxidase
CCS: copper chaperone for superoxide dismutase
CI: copper ionophore
COMMD3: copper metabolizing MURR1 domain 3
COX17: cytochrome c oxidase 17
CP: ceruloplasmin
CRG: cuproptosis-related gene
CTR1: copper transport protein 1
DAMP: damage-associated molecular pattern
DCYTB: duodenal cytochrome b
DSF: disulfiram
DLAT: dihydrolipoyl transacetylase
DLST: dihydrolipoyllysine-residue succinyltransferase
ES: elesclomol
FDX1: ferredoxin 1
Fe-S: iron-sulfur
GAPDH: glyceraldehyde-3-phosphate dehydrogenase
GCSH: glycine cleavage system protein H
GPX4: glutathione peroxidase 4
GSH: glutathione
GSSG: glutathione disulfide
HCC: hepatocellular carcinoma
HIF-1α: hypoxia-inducible factor-1α
HIR: hepatic ischemia-reperfusion
IEC: intestinal epithelial cell
LIAS: lipoic acid synthase
LIPT1/2: lipoyltransferase 1/2
MAFLD: metabolic dysfunction-associated fatty liver disease
MAPK: mitogen-activated protein kinase
MD: Menkes disease
MELK: maternal embryonic leucine zipper kinase
MMP: matrix metalloproteinase
MOXD1: monooxygenase DBH-like 1
MT-1/2: metallothionein 1/2
mtETC: mitochondrial electron transfer chain
NAFLD: non-alcoholic fatty liver disease
NASH: non-alcoholic steatohepatitis
NF-κB: nuclear factor-kappa B
PARP1: Poly (ADP-ribose) polymerase 1
PCD: programmed cell death
PDHX: pyruvate dehydrogenase complex component X
PD-L1: programmed death ligand-1
PI3K: phosphoinositide-3-kinase
PXR: pregnane X receptor
ROS: reactive oxygen species
SOD1: superoxide dismutase 1
SSAO: semicarbazide-sensitive amine oxidase
STEAP: six transmembrane epithelial antigen of the prostate
TCA: tricarboxylic acid
TME: tumor microenvironment
TTM: tetrathiomolybdate
VEGF: vascular endothelial growth factor
WD: Wilson disease
ZnT1: zinc transporter 1

## References

[1] Yang L Yang P Lip GYH Ren J. Copper homeostasis and cuproptosis in cardiovascular disease therapeutics. Trends Pharmacol Sci. (2023) 44:573–85. doi: 10.1016/j.tips.2023.07.004, PMID: 37500296

[2] Chen H Han Z Luo Q Wang Y Li Q Zhou L. Radiotherapy modulates tumor cell fate decisions: A review. Radiat Oncol (London England). (2022) 17:196. doi: 10.1186/s13014-022-02171-7, PMID: 36457125 PMC9714175

[3] Tsvetkov P Coy S Petrova B Dreishpoon M Verma A Abdusamad M. Copper induces cell death by targeting lipoylated TCA cycle proteins. Sci (New York NY). (2022) 375:1254–61. doi: 10.1126/science.abf0529, PMID: 35298263 PMC9273333

[4] Wang Y Zhang L Zhou F. Cuproptosis: A new form of programmed cell death. Cell Mol Immunol. (2022) 19:867–8. doi: 10.1038/s41423-022-00866-1, PMID: 35459854 PMC9338229

[5] Cobine PA Brady DC. Cuproptosis: cellular and molecular mechanisms underlying copper-induced cell death. Mol Cell. (2022) 82:1786–7. doi: 10.1016/j.molcel.2022.05.001, PMID: 35594843

[6] Jawed R Bhatti H. Cuproptosis in lung cancer: therapeutic options and prognostic models. Apoptosis: an Int J programmed Cell Death. (2024) 29:1393–8. doi: 10.1007/s10495-024-01978-x, PMID: 38735011

[7] Chen L Min J Wang F. Copper homeostasis and cuproptosis in health and disease. Signal transduction targeted Ther. (2022) 7:378. doi: 10.1038/s41392-022-01229-y, PMID: 36414625 PMC9681860

[8] Xie J Yang Y Gao Y He J. Cuproptosis: mechanisms and links with cancers. Mol Cancer. (2023) 22:46. doi: 10.1186/s12943-023-01732-y, PMID: 36882769 PMC9990368

[9] Liu H. Pan-cancer profiles of the cuproptosis gene set. Am J Cancer Res. (2022) 12:4074–81. doi: 10.21203/rs.3.rs-1716214/v1 PMC9442004 36119826

[10] Liu H Tang T. Pan-cancer genetic analysis of cuproptosis and copper metabolism-related gene set. Front Oncol. (2022) 12:952290. doi: 10.3389/fonc.2022.952290, PMID: 36276096 PMC9582932

[11] Liu H. Expression and potential immune involvement of cuproptosis in kidney renal clear cell carcinoma. Cancer Genet. (2023) 274-275:21–5. doi: 10.1016/j.cancergen.2023.03.002, PMID: 36963335

[12] Yang Q Liu X Tang H Chen Y Bai L. Emerging roles of cuproptosis in liver diseases. Digestive liver disease: Off J Ital Soc Gastroenterol Ital Assoc Study Liver. (2025). doi: 10.1016/j.dld.2025.04.011, PMID: 40254494

[13] Blades B Ayton S Hung YH Bush AI La Fontaine S. Copper and lipid metabolism: A reciprocal relationship. Biochim Biophys Acta Gen Subj. (2021) 1865:129979. doi: 10.1016/j.bbagen.2021.129979, PMID: 34364973

[14] Wyman S Simpson RJ McKie AT Sharp PA. Dcytb (Cybrd1) functions as both a ferric and a cupric reductase *in vitro* . FEBS Lett. (2008) 582:1901–6. doi: 10.1016/j.febslet.2008.05.010, PMID: 18498772

[15] Scarl RT Lawrence CM Gordon HM Nunemaker CS. STEAP4: its emerging role in metabolism and homeostasis of cellular iron and copper. J Endocrinol. (2017) 234:R123–r34. doi: 10.1530/joe-16-0594, PMID: 28576871 PMC6166870

[16] Su Y Zhang X Li S Xie W Guo J. Emerging roles of the copper-CTR1 axis in tumorigenesis. Mol Cancer research: MCR. (2022) 20:1339–53. doi: 10.1158/1541-7786.Mcr-22-0056, PMID: 35604085

[17] Parrish A Boudaud M Kuehn A Ollert M Desai MS. Intestinal mucus barrier: A missing piece of the puzzle in food allergy. Trends Mol Med. (2022) 28:36–50. doi: 10.1016/j.molmed.2021.10.004, PMID: 34810087

[18] Reznik N Gallo AD Rush KW Javitt G Fridmann-Sirkis Y Ilani T. Intestinal mucin is a chaperone of multivalent copper. Cell. (2022) 185:4206–15.e11. doi: 10.1016/j.cell.2022.09.021, PMID: 36206754

[19] Hoogenraad TU Van Hattum J Van den Hamer CJ. Management of Wilson’s disease with zinc sulphate. Experience in a series of 27 patients. J neurological Sci. (1987) 77:137–46. doi: 10.1016/0022-510x(87)90116-x, PMID: 3819764

[20] Chen Y Li Z Zhang H Chen H Hao J Liu H. Mitochondrial metabolism and targeted treatment strategies in ischemic-induced acute kidney injury. Cell Death Discov. (2024) 10:69. doi: 10.1038/s41420-024-01843-5, PMID: 38341438 PMC10858869

[21] Kaler SG. Translational research investigations on atp7a: an important human copper ATPase. Ann New York Acad Sci. (2014) 1314:64–8. doi: 10.1111/nyas.12422, PMID: 24735419 PMC4095951

[22] Ramos D Mar D Ishida M Vargas R Gaite M Montgomery A. Mechanism of copper uptake from blood plasma ceruloplasmin by mammalian cells. PloS One. (2016) 11:e0149516. doi: 10.1371/journal.pone.0149516, PMID: 26934375 PMC4774968

[23] Yamaguchi Y Heiny ME Gitlin JD. Isolation and characterization of a human liver cDNA as a candidate gene for Wilson disease. Biochem Biophys Res Commun. (1993) 197:271–7. doi: 10.1006/bbrc.1993.2471, PMID: 8250934

[24] Hung IH Suzuki M Yamaguchi Y Yuan DS Klausner RD Gitlin JD. Biochemical characterization of the Wilson disease protein and functional expression in the yeast saccharomyces cerevisiae. J Biol Chem. (1997) 272:21461–6. doi: 10.1074/jbc.272.34.21461, PMID: 9261163

[25] Tanzi RE Petrukhin K Chernov I Pellequer JL Wasco W Ross B. The Wilson disease gene is a copper transporting ATPase with homology to the Menkes disease gene. Nat Genet. (1993) 5:344–50. doi: 10.1038/ng1293-344, PMID: 8298641

[26] Wee NK Weinstein DC Fraser ST Assinder SJ. The mammalian copper transporters CTR1 and CTR2 and their roles in development and disease. Int J Biochem Cell Biol. (2013) 45:960–3. doi: 10.1016/j.biocel.2013.01.018, PMID: 23391749

[27] Lerch K. Copper metallothionein, a copper-binding protein from neurospora crassa. Nature. (1980) 284:368–70. doi: 10.1038/284368a0, PMID: 6444697

[28] Huang D Chen L Ji Q Xiang Y Zhou Q Chen K. Lead aggravates Alzheimer’s disease pathology via mitochondrial copper accumulation regulated by COX17. Redox Biol. (2024) 69:102990. doi: 10.1016/j.redox.2023.102990, PMID: 38091880 PMC10716782

[29] Boyd SD Ullrich MS Skopp A Winkler DD. Copper sources for Sod1 activation. Antioxidants (Basel Switzerland). (2020) 9:500. doi: 10.3390/antiox9060500, PMID: 32517371 PMC7346115

[30] Muller PA Klomp LW. ATOX1: A novel copper-responsive transcription factor in mammals? Int J Biochem Cell Biol. (2009) 41:1233–6. doi: 10.1016/j.biocel.2008.08.001, PMID: 18761103

[31] Wang Y Zhang B Fan F Zhao F Xu J Zheng Y. COMMD3 regulates copper metabolism via the ATOX1-ATP7A-LOX axis to promote multiple myeloma progression. Biomedicines. (2025) 13:351. doi: 10.3390/biomedicines13020351, PMID: 40002764 PMC11852399

[32] Pufahl RA Singer CP Peariso KL Lin SJ Schmidt PJ Fahrni CJ. Metal ion chaperone function of the soluble Cu(I) receptor Atx1. Sci (New York NY). (1997) 278:853–6. doi: 10.1126/science.278.5339.853, PMID: 9346482

[33] Lutsenko S. Human copper homeostasis: A network of interconnected pathways. Curr Opin Chem Biol. (2010) 14:211–7. doi: 10.1016/j.cbpa.2010.01.003, PMID: 20117961 PMC6365103

[34] Halliwell B Gutteridge JM. Oxygen toxicity, oxygen radicals, transition metals and disease. Biochem J. (1984) 219:1–14. doi: 10.1042/bj2190001, PMID: 6326753 PMC1153442

[35] Mariño Z Schilsky ML. Wilson disease: novel diagnostic and therapeutic approaches. Semin liver Dis. (2025) 45:221–35. doi: 10.1055/a-2460-8999, PMID: 39496313 PMC12962356

[36] Scheinberg IH Sternlieb I. Wilson disease and idiopathic copper toxicosis. Am J Clin Nutr. (1996) 63:842s–5s. doi: 10.1093/ajcn/63.5.842, PMID: 8615372

[37] Oliveri V. Selective targeting of cancer cells by copper ionophores: an overview. Front Mol Biosci. (2022) 9:841814. doi: 10.3389/fmolb.2022.841814, PMID: 35309510 PMC8931543

[38] Allensworth JL Evans MK Bertucci F Aldrich AJ Festa RA Finetti P. Disulfiram (DSF) acts as a copper ionophore to induce copper-dependent oxidative stress and mediate anti-tumor efficacy in inflammatory breast cancer. Mol Oncol. (2015) 9:1155–68. doi: 10.1016/j.molonc.2015.02.007, PMID: 25769405 PMC4493866

[39] Kirshner JR He S Balasubramanyam V Kepros J Yang CY Zhang M. Elesclomol induces cancer cell apoptosis through oxidative stress. Mol Cancer Ther. (2008) 7:2319–27. doi: 10.1158/1535-7163.Mct-08-0298, PMID: 18723479

[40] Nagai M Vo NH Shin Ogawa L Chimmanamada D Inoue T Chu J. The oncology drug elesclomol selectively transports copper to the mitochondria to induce oxidative stress in cancer cells. Free Radical Biol Med. (2012) 52:2142–50. doi: 10.1016/j.freeradbiomed.2012.03.017, PMID: 22542443

[41] Tsvetkov P Detappe A Cai K Keys HR Brune Z Ying W. Mitochondrial metabolism promotes adaptation to proteotoxic stress. Nat Chem Biol. (2019) 15:681–9. doi: 10.1038/s41589-019-0291-9, PMID: 31133756 PMC8183600

[42] Tsvetkov P Detappe A Cai K Keys HR Brune Z Ying W. Author correction: mitochondrial metabolism promotes adaptation to proteotoxic stress. Nat Chem Biol. (2019) 15:757. doi: 10.1038/s41589-019-0315-5, PMID: 31164776 PMC8189136

[43] Yang W Wang Y Huang Y Yu J Wang T Li C. 4-octyl itaconate inhibits aerobic glycolysis by targeting GAPDH to promote cuproptosis in colorectal cancer. Biomedicine pharmacotherapy = Biomedecine pharmacotherapie. (2023) 159:114301. doi: 10.1016/j.biopha.2023.114301, PMID: 36706634

[44] Wang D Tian Z Zhang P Zhen L Meng Q Sun B. The molecular mechanisms of cuproptosis and its relevance to cardiovascular disease. Biomedicine pharmacotherapy = Biomedecine pharmacotherapie. (2023) 163:114830. doi: 10.1016/j.biopha.2023.114830, PMID: 37150036

[45] Rowland EA Snowden CK Cristea IM. Protein lipoylation: an evolutionarily conserved metabolic regulator of health and disease. Curr Opin Chem Biol. (2018) 42:76–85. doi: 10.1016/j.cbpa.2017.11.003, PMID: 29169048 PMC5965299

[46] Hultberg B Andersson A Isaksson A. Copper ions differ from other thiol reactive metal ions in their effects on the concentration and redox status of thiols in hela cell cultures. Toxicology. (1997) 117:89–97. doi: 10.1016/s0300-483x(96)03554-8, PMID: 9057888

[47] Solmonson A DeBerardinis RJ. Lipoic acid metabolism and mitochondrial redox regulation. J Biol Chem. (2018) 293:7522–30. doi: 10.1074/jbc.TM117.000259, PMID: 29191830 PMC5961061

[48] Gao F Yuan Y Ding Y Li PY Chang Y He XX. DLAT as a cuproptosis promoter and a molecular target of elesclomol in hepatocellular carcinoma. Curr Med Sci. (2023) 43:526–38. doi: 10.1007/s11596-023-2755-0, PMID: 37286711

[49] Bernardinelli E Costa R Scantamburlo G To J Morabito R Nofziger C. Mis-targeting of the mitochondrial protein LIPT2 leads to apoptotic cell death. PloS One. (2017) 12:e0179591. doi: 10.1371/journal.pone.0179591, PMID: 28628643 PMC5476274

[50] Cicchillo RM Iwig DF Jones AD Nesbitt NM Baleanu-Gogonea C Souder MG. Lipoyl synthase requires two equivalents of S-adenosyl-L-methionine to synthesize one equivalent of lipoic acid. Biochemistry. (2004) 43:6378–86. doi: 10.1021/bi049528x, PMID: 15157071

[51] Ni M Solmonson A Pan C Yang C Li D Notzon A. Functional assessment of lipoyltransferase-1 deficiency in cells, mice, and humans. Cell Rep. (2019) 27:1376–86.e6. doi: 10.1016/j.celrep.2019.04.005, PMID: 31042466 PMC7351313

[52] Lin CH Chin Y Zhou M Sobol RW Hung MC Tan M. Protein lipoylation: mitochondria, cuproptosis, and beyond. Trends Biochem Sci. (2024) 49:729–44. doi: 10.1016/j.tibs.2024.04.002, PMID: 38714376

[53] Qin Y Liu Y Xiang X Long X Chen Z Huang X. Cuproptosis correlates with immunosuppressive tumor microenvironment based on pan-cancer multiomics and single-cell sequencing analysis. Mol Cancer. (2023) 22:59. doi: 10.1186/s12943-023-01752-8, PMID: 36959665 PMC10037895

[54] Zhang B Xie L Liu J Liu A He M. Construction and validation of a cuproptosis-related prognostic model for glioblastoma. Front Immunol. (2023) 14:1082974. doi: 10.3389/fimmu.2023.1082974, PMID: 36814929 PMC9939522

[55] Hall DO Cammack R Rao KK. Role for ferredoxins in the origin of life and biological evolution. Nature. (1971) 233:136–8. doi: 10.1038/233136a0, PMID: 12058758

[56] Dreishpoon MB Bick NR Petrova B Warui DM Cameron A Booker SJ. FDX1 regulates cellular protein lipoylation through direct binding to lias. J Biol Chem. (2023) 299:105046. doi: 10.1016/j.jbc.2023.105046, PMID: 37453661 PMC10462841

[57] Pan TT Huang JY Wang XD Chen DZ Chen YP. Copper’s dual role: reviewing its impact on liver health and disease. Int Immunopharmacol. (2025) 152:114391. doi: 10.1016/j.intimp.2025.114391, PMID: 40073812

[58] Lu J Liu X Li X Li H Shi L Xia X. Copper regulates the host innate immune response against bacterial infection via activation of ALPK1 kinase. Proc Natl Acad Sci United States America. (2024) 121:e2311630121. doi: 10.1073/pnas.2311630121, PMID: 38232278 PMC10823219

[59] Djoko KY Ong CL Walker MJ McEwan AG. The role of copper and zinc toxicity in innate immune defense against bacterial pathogens. J Biol Chem. (2015) 290:18954–61. doi: 10.1074/jbc.R115.647099, PMID: 26055706 PMC4521016

[60] Yu L Yousuf S Yousuf S Yeh J Biggins SW Morishima C. Copper deficiency is an independent risk factor for mortality in patients with advanced liver disease. Hepatol Commun. (2023) 7:e0076. doi: 10.1097/hc9.0000000000000076, PMID: 36809345 PMC9949837

[61] Li L Lv L Wang Z Liu X Wang Q Zhu H. From copper homeostasis to cuproptosis: A new perspective on CNS immune regulation and neurodegenerative diseases. Front Neurol. (2025) 16:1581045. doi: 10.3389/fneur.2025.1581045, PMID: 40510202 PMC12158703

[62] Wang Y Ding Y Yao D Dong H Ji C Wu J. Copper-based nanoscale coordination polymers augmented tumor radioimmunotherapy for immunogenic cell death induction and T-cell infiltration. Small (Weinheim an der Bergstrasse Germany). (2021) 17:e2006231. doi: 10.1002/smll.202006231, PMID: 33522120

[63] Mao Y Chen H Zhu W Ni S Luo S Tang S. Cuproptosis cell death molecular events and pathways to liver disease. J Inflammation Res. (2025) 18:883–94. doi: 10.2147/jir.S498340, PMID: 39867947 PMC11760270

[64] Cheng F Peng G Lu Y Wang K Ju Q Ju Y. Relationship between copper and immunity: the potential role of copper in tumor immunity. Front Oncol. (2022) 12:1019153. doi: 10.3389/fonc.2022.1019153, PMID: 36419894 PMC9676660

[65] Tümer Z Møller LB. Menkes disease. Eur J Hum genetics: EJHG. (2010) 18:511–8. doi: 10.1038/ejhg.2009.187, PMID: 19888294 PMC2987322

[66] Ryan A Twomey PJ Cook P. Wilson’s disease: best practice. J Clin Pathol. (2023) 76:435–41. doi: 10.1136/jcp-2022-208551, PMID: 37045587

[67] Yang X Tang XP Zhang YH Luo KZ Jiang YF Luo HY. Prospective evaluation of the diagnostic accuracy of hepatic copper content, as determined using the entire core of a liver biopsy sample. Hepatol (Baltimore Md). (2015) 62:1731–41. doi: 10.1002/hep.27932, PMID: 26095812 PMC4744736

[68] Gromadzka G Wierzbicka D Litwin T Przybyłkowski A. Iron metabolism is disturbed and anti-copper treatment improves but does not normalize iron metabolism in Wilson’s disease. Biometals: an Int J role metal ions biology biochemistry Med. (2021) 34:407–14. doi: 10.1007/s10534-021-00289-x, PMID: 33555495 PMC7940312

[69] La Fontaine S Ackland ML Mercer JF. Mammalian copper-transporting P-type ATPases, ATP7A and ATP7B: emerging roles. Int J Biochem Cell Biol. (2010) 42:206–9. doi: 10.1016/j.biocel.2009.11.007, PMID: 19922814 PMC2846448

[70] Sternlieb I. Fraternal concordance of types of abnormal hepatocellular mitochondria in Wilson’s disease. Hepatol (Baltimore Md). (1992) 16:728–32. doi: 10.1002/hep.1840160319, PMID: 1505917

[71] Zischka H Lichtmannegger J Schmitt S Jägemann N Schulz S Wartini D. Liver mitochondrial membrane crosslinking and destruction in a rat model of Wilson disease. J Clin Invest. (2011) 121:1508–18. doi: 10.1172/jci45401, PMID: 21364284 PMC3068979

[72] Lichtmannegger J Leitzinger C Wimmer R Schmitt S Schulz S Kabiri Y. Methanobactin reverses acute liver failure in a rat model of Wilson disease. J Clin Invest. (2016) 126:2721–35. doi: 10.1172/jci85226, PMID: 27322060 PMC4922707

[73] Borchard S Bork F Rieder T Eberhagen C Popper B Lichtmannegger J. The exceptional sensitivity of brain mitochondria to copper. Toxicol vitro: an Int J published Assoc BIBRA. (2018) 51:11–22. doi: 10.1016/j.tiv.2018.04.012, PMID: 29715505

[74] Gu M Cooper JM Butler P Walker AP Mistry PK Dooley JS. Oxidative-phosphorylation defects in liver of patients with Wilson’s disease. Lancet (London England). (2000) 356:469–74. doi: 10.1016/s0140-6736(00)02556-3, PMID: 10981891

[75] Sauer SW Merle U Opp S Haas D Hoffmann GF Stremmel W. Severe dysfunction of respiratory chain and cholesterol metabolism in Atp7b(-/-) mice as a model for Wilson disease. Biochim Biophys Acta. (2011) 1812:1607–15. doi: 10.1016/j.bbadis.2011.08.011, PMID: 21920437

[76] Russell K Gillanders LK Orr DW Plank LD. Dietary copper restriction in Wilson’s disease. Eur J Clin Nutr. (2018) 72:326–31. doi: 10.1038/s41430-017-0002-0, PMID: 29235558

[77] Weiss KH Thurik F Gotthardt DN Schäfer M Teufel U Wiegand F. Efficacy and safety of oral chelators in treatment of patients with Wilson disease. Clin Gastroenterol hepatology: Off Clin Pract J Am Gastroenterological Assoc. (2013) 11:1028–35. doi: 10.1016/j.cgh.2013.03.012, PMID: 23542331

[78] Van Caillie-Bertrand M Degenhart HJ Luijendijk I Bouquet J Sinaasappel M. Wilson’s disease: assessment of D-penicillamine treatment. Arch Dis childhood. (1985) 60:652–5. doi: 10.1136/adc.60.7.652, PMID: 4026361 PMC1777273

[79] Kumar S Patra BR Irtaza M Rao PK Giri S Darak H. Adverse events with D-penicillamine therapy in hepatic Wilson’s disease: A single-center retrospective audit. Clin Drug Invest. (2022) 42:177–84. doi: 10.1007/s40261-022-01117-x, PMID: 35102516

[80] Schilsky ML Czlonkowska A Zuin M Cassiman D Twardowschy C Poujois A. Trientine tetrahydrochloride versus penicillamine for maintenance therapy in Wilson disease (Chelate): A randomised, open-label, non-inferiority, phase 3 trial. Lancet Gastroenterol Hepatol. (2022) 7:1092–102. doi: 10.1016/s2468-1253(22)00270-9, PMID: 36183738

[81] Kirk FT Munk DE Swenson ES Quicquaro AM Vendelbo MH Larsen A. Effects of tetrathiomolybdate on copper metabolism in healthy volunteers and in patients with Wilson disease. J Hepatol. (2024) 80:586–95. doi: 10.1016/j.jhep.2023.11.023, PMID: 38081365

[82] Weiss KH Askari FK Czlonkowska A Ferenci P Bronstein JM Bega D. Bis-choline tetrathiomolybdate in patients with Wilson’s disease: an open-label, multicentre, phase 2 study. Lancet Gastroenterol Hepatol. (2017) 2:869–76. doi: 10.1016/s2468-1253(17)30293-5, PMID: 28988934

[83] Guo YY Liang NN Zhang XY Ren YH Wu WZ Liu ZB. Mitochondrial GPX4 acetylation is involved in cadmium-induced renal cell ferroptosis. Redox Biol. (2024) 73:103179. doi: 10.1016/j.redox.2024.103179, PMID: 38733909 PMC11103486

[84] Errichiello F Picariello L Forino M Blaiotta G Petruzziello E Moio L. Copper (II) level in musts affects acetaldehyde concentration, phenolic composition, and chromatic characteristics of red and white wines. Molecules (Basel Switzerland). (2024) 29:2907. doi: 10.3390/molecules29122907, PMID: 38930972 PMC11206618

[85] Lin H Chen D Du Q Pan T Tu H Xu Y. Dietary copper plays an important role in maintaining intestinal barrier integrity during alcohol-induced liver disease through regulation of the intestinal HIF-1α Signaling pathway and oxidative stress. Front Physiol. (2020) 11:369. doi: 10.3389/fphys.2020.00369, PMID: 32457642 PMC7227433

[86] Shao T Zhao C Li F Gu Z Liu L Zhang L. Intestinal hif-1α Deletion exacerbates alcoholic liver disease by inducing intestinal dysbiosis and barrier dysfunction. J Hepatol. (2018) 69:886–95. doi: 10.1016/j.jhep.2018.05.021, PMID: 29803899 PMC6615474

[87] Sun X Wang J Ge Q Li C Ma T Fang Y. Interactive effects of copper and functional substances in wine on alcoholic hepatic injury in mice. Foods (Basel Switzerland). (2022) 11:2383. doi: 10.3390/foods11162383, PMID: 36010383 PMC9407149

[88] Yu W Liao J Yang F Zhang H Chang X Yang Y. Chronic tribasic copper chloride exposure induces rat liver damage by disrupting the mitophagy and apoptosis pathways. Ecotoxicology Environ Saf. (2021) 212:111968. doi: 10.1016/j.ecoenv.2021.111968, PMID: 33550083

[89] Wang W Hua S Li J Zhao J Zhang Y Jiang J. Tumour microenvironment landscape and immunotherapy response in bladder cancer decoded by stromal MOXD1 based on copper-related genes signature. Front Oncol. (2022) 12:1081091. doi: 10.3389/fonc.2022.1081091, PMID: 36620542 PMC9815449

[90] Pan T Zhao Z Lu J Wen H Zhang J Xu Y. Fenofibrate inhibits MOXD1 and PDZK1IP1 expression and improves lipid deposition and inflammation in mice with alcoholic fatty liver. Life Sci. (2024) 336:122321. doi: 10.1016/j.lfs.2023.122321, PMID: 38042280

[91] Hou S Wang D Yuan X Yuan X Yuan Q. Identification of biomarkers co-associated with M1 macrophages, ferroptosis and cuproptosis in alcoholic hepatitis by bioinformatics and experimental verification. Front Immunol. (2023) 14:1146693. doi: 10.3389/fimmu.2023.1146693, PMID: 37090703 PMC10117880

[92] Pan T Wu S Wang S Wang X Chen D Chen Y. Novel insights into cuproptosis in alcoholic liver disease using bioinformatics analysis and experimental validation. Int Immunopharmacol. (2025) 146:113828. doi: 10.1016/j.intimp.2024.113828, PMID: 39709914

[93] Zeng C Lin J Zhang K Ou H Shen K Liu Q. SHARPIN promotes cell proliferation of cholangiocarcinoma and inhibits ferroptosis via P53/SLC7A11/GPX4 signaling. Cancer Sci. (2022) 113:3766–75. doi: 10.1111/cas.15531, PMID: 35968603 PMC9633309

[94] Liu G Chen X. The ferredoxin reductase gene is regulated by the P53 family and sensitizes cells to oxidative stress-induced apoptosis. Oncogene. (2002) 21:7195–204. doi: 10.1038/sj.onc.1205862, PMID: 12370809

[95] Zawacka-Pankau J Grinkevich VV Hünten S Nikulenkov F Gluch A Li H. Inhibition of glycolytic enzymes mediated by pharmacologically activated P53: targeting warburg effect to fight cancer. J Biol Chem. (2011) 286:41600–15. doi: 10.1074/jbc.M111.240812, PMID: 21862591 PMC3308870

[96] Guo X Yin X Liu Z Wang J. Non-alcoholic fatty liver disease (NAFLD) pathogenesis and natural products for prevention and treatment. Int J Mol Sci. (2022) 23:15489. doi: 10.3390/ijms232415489, PMID: 36555127 PMC9779435

[97] Leoni S Tovoli F Napoli L Serio I Ferri S Bolondi L. Current guidelines for the management of non-alcoholic fatty liver disease: A systematic review with comparative analysis. World J Gastroenterol. (2018) 24:3361–73. doi: 10.3748/wjg.v24.i30.3361, PMID: 30122876 PMC6092580

[98] Antonucci L Porcu C Iannucci G Balsano C Barbaro B. Non-alcoholic fatty liver disease and nutritional implications: special focus on copper. Nutrients. (2017) 9:1137. doi: 10.3390/nu9101137, PMID: 29057834 PMC5691753

[99] Golabi P Owrangi S Younossi ZM. Global perspective on nonalcoholic fatty liver disease and nonalcoholic steatohepatitis - prevalence, clinical impact, economic implications and management strategies. Alimentary Pharmacol Ther. (2024) 59 Suppl 1:S1–s9. doi: 10.1111/apt.17833, PMID: 38813821

[100] Eslam M Newsome PN Sarin SK Anstee QM Targher G Romero-Gomez M. A new definition for metabolic dysfunction-associated fatty liver disease: an international expert consensus statement. J Hepatol. (2020) 73:202–9. doi: 10.1016/j.jhep.2020.03.039, PMID: 32278004

[101] Li Y Qi P Song SY Wang Y Wang H Cao P. Elucidating cuproptosis in metabolic dysfunction-associated steatotic liver disease. Biomedicine pharmacotherapy = Biomedecine pharmacotherapie. (2024) 174:116585. doi: 10.1016/j.biopha.2024.116585, PMID: 38615611

[102] Tan W Zhang J Chen L Wang Y Chen R Zhang H. Copper homeostasis and cuproptosis-related genes: therapeutic perspectives in non-alcoholic fatty liver disease. Diabetes Obes Metab. (2024) 26:4830–45. doi: 10.1111/dom.15846, PMID: 39233500

[103] Li L Yi Y Shu X Li J Kang H Chang Y. The correlation between serum copper and non-alcoholic fatty liver disease in American adults: an analysis based on NHANES 2011 to 2016. Biol Trace element Res. (2024) 202:4398–409. doi: 10.1007/s12011-023-04029-9, PMID: 38168830

[104] Lan Y Wu S Wang Y Chen S Liao W Zhang X. Association between blood copper and nonalcoholic fatty liver disease according to sex. Clin Nutr (Edinburgh Scotland). (2021) 40:2045–52. doi: 10.1016/j.clnu.2020.09.026, PMID: 33039155

[105] Chen Y Wu C Li G Wang W Tang S. Comparison of copper concentration between non-alcoholic fatty liver disease patients and normal individuals: A meta-analysis. Front Public Health. (2023) 11:1095916. doi: 10.3389/fpubh.2023.1095916, PMID: 36817887 PMC9929538

[106] Jiang M Tao X Pang Y Qin Z Song E Song Y. Copper oxide nanoparticles induce non-alcoholic fatty liver disease by disrupting bile acid homeostasis and perturbing the intestinal microbial homeostasis. J hazardous materials. (2024) 480:136416. doi: 10.1016/j.jhazmat.2024.136416, PMID: 39531819

[107] Barretto SA Lasserre F Huillet M Régnier M Polizzi A Lippi Y. The pregnane X receptor drives sexually dimorphic hepatic changes in lipid and xenobiotic metabolism in response to gut microbiota in mice. Microbiome. (2021) 9:93. doi: 10.1186/s40168-021-01050-9, PMID: 33879258 PMC8059225

[108] Liu H Guo H Deng H Cui H Fang J Zuo Z. Copper induces hepatic inflammatory responses by activation of MAPKs and NF-κB signalling pathways in the mouse. Ecotoxicology Environ Saf. (2020) 201:110806. doi: 10.1016/j.ecoenv.2020.110806, PMID: 32512418

[109] Aigner E Theurl I Haufe H Seifert M Hohla F Scharinger L. Copper availability contributes to iron perturbations in human nonalcoholic fatty liver disease. Gastroenterology. (2008) 135:680–8. doi: 10.1053/j.gastro.2008.04.007, PMID: 18505688

[110] Ma C Han L Zhu Z Heng Pang C Pan G. Mineral metabolism and ferroptosis in non-alcoholic fatty liver diseases. Biochem Pharmacol. (2022) 205:115242. doi: 10.1016/j.bcp.2022.115242, PMID: 36084708

[111] Millo H Werman MJ. Hepatic fructose-metabolizing enzymes and related metabolites: role of dietary copper and gender. J Nutr Biochem. (2000) 11:374–81. doi: 10.1016/s0955-2863(00)00093-0, PMID: 11044632

[112] Song M Vos MB McClain CJ. Copper-fructose interactions: A novel mechanism in the pathogenesis of NAFLD. Nutrients. (2018) 10:1815. doi: 10.3390/nu10111815, PMID: 30469339 PMC6266129

[113] Tamura Y. The role of zinc homeostasis in the prevention of diabetes mellitus and cardiovascular diseases. J Atheroscl Thromb. (2021) 28:1109–22. doi: 10.5551/jat.RV17057, PMID: 34148917 PMC8592709

[114] Gembillo G Labbozzetta V Giuffrida AE Peritore L Calabrese V Spinella C. Potential role of copper in diabetes and diabetic kidney disease. Metabolites. (2022) 13:17. doi: 10.3390/metabo13010017, PMID: 36676942 PMC9866181

[115] Newsome PN Sanyal AJ Neff G Schattenberg JM Ratziu V Ertle J. A randomised phase IIa trial of amine oxidase copper-containing 3 (AOC3) inhibitor BI 1467335 in adults with non-alcoholic steatohepatitis. Nat Commun. (2023) 14:7151. doi: 10.1038/s41467-023-42398-w, PMID: 37932258 PMC10628239

[116] Obata T. Diabetes and semicarbazide-sensitive amine oxidase (SSAO) activity: A review. Life Sci. (2006) 79:417–22. doi: 10.1016/j.lfs.2006.01.017, PMID: 16487546

[117] Yang H Liu CN Wolf RM Ralle M Dev S Pierson H. Obesity is associated with copper elevation in serum and tissues. Metallomics: integrated biometal Sci. (2019) 11:1363–71. doi: 10.1039/c9mt00148d, PMID: 31249997 PMC7753954

[118] Xu G Yan T Peng Q Li H Wu W Yi X. Overexpression of the lias gene attenuates hepatic steatosis in Leprdb/db mice. J Endocrinol. (2021) 248:119–31. doi: 10.1530/joe-19-0606, PMID: 33263565

[119] Zhao Z Luan T Wan J Du H Hu J Liu H. Elucidating cuproptosis-associated genes in the progression from Nash to HCC using bulk and single-cell RNA sequencing analyses and experimental validation. Medicina (Kaunas Lithuania). (2023) 59:1639. doi: 10.3390/medicina59091639, PMID: 37763758 PMC10536385

[120] Ouyang G Wu Z Liu Z Pan G Wang Y Liu J. Identification and validation of potential diagnostic signature and immune cell infiltration for NAFLD based on cuproptosis-related genes by bioinformatics analysis and machine learning. Front Immunol. (2023) 14:1251750. doi: 10.3389/fimmu.2023.1251750, PMID: 37822923 PMC10562635

[121] Li J Zhang Y Ma X Liu R Xu C He Q. Identification and validation of cuproptosis-related genes for diagnosis and therapy in nonalcoholic fatty liver disease. Mol Cell Biochem. (2025) 480:473–89. doi: 10.1007/s11010-024-04957-7, PMID: 38512536

[122] Sarin SK Choudhury A Sharma MK Maiwall R Al Mahtab M Rahman S. Acute-on-chronic liver failure: consensus recommendations of the Asian pacific association for the study of the liver (APASL): an update. Hepatol Int. (2019) 13:353–90. doi: 10.1007/s12072-019-09946-3, PMID: 31172417 PMC6728300

[123] Jantsch W Kulig K Rumack BH. Massive copper sulfate ingestion resulting in hepatotoxicity. J Toxicol Clin Toxicol. (1984) 22:585–8. doi: 10.3109/15563658408992588, PMID: 6535851

[124] Sinkovic A Strdin A Svensek F. Severe acute copper sulphate poisoning: A case report. Arhiv za higijenu rada i toksikologiju. (2008) 59:31–5. doi: 10.2478/10004-1254-59-2008-1847, PMID: 18407869

[125] de Oliveira THC Gonçalves GKN. Liver ischemia reperfusion injury: mechanisms, cellular pathways, and therapeutic approaches. Int Immunopharmacol. (2025) 150:114299. doi: 10.1016/j.intimp.2025.114299, PMID: 39961215

[126] Cai X Deng J Zhou X Wang K Cai H Yan Y. Comprehensive analysis of cuproptosis-related genes involved in immune infiltration and their use in the diagnosis of hepatic ischemia-reperfusion injury: an experimental study. Int J Surg (London England). (2025) 111:242–56. doi: 10.1097/js9.0000000000001893, PMID: 38935114 PMC11745764

[127] Guo Z Liu J Liang G Liang H Zhong M Tomlinson S. Identification and validation of cuproptosis-related genes in acetaminophen-induced liver injury using bioinformatics analysis and machine learning. Front Immunol. (2024) 15:1371446. doi: 10.3389/fimmu.2024.1371446, PMID: 38994365 PMC11236684

[128] Luo X Linghu M Zhou X Ru Y Huang Q Liu D. Merestinib inhibits cuproptosis by targeting NRF2 to alleviate acute liver injury. Free Radical Biol Med. (2025) 229:68–81. doi: 10.1016/j.freeradbiomed.2025.01.029, PMID: 39824447

[129] Huang Y Zhang Y Lin Z Han M Cheng H. Altered serum copper homeostasis suggests higher oxidative stress and lower antioxidant capability in patients with chronic hepatitis B. Medicine. (2018) 97:e11137. doi: 10.1097/md.0000000000011137, PMID: 29901643 PMC6023651

[130] Yu L Liou IW Biggins SW Yeh M Jalikis F Chan LN. Copper deficiency in liver diseases: A case series and pathophysiological considerations. Hepatol Commun. (2019) 3:1159–65. doi: 10.1002/hep4.1393, PMID: 31388635 PMC6671688

[131] Hatano R Ebara M Fukuda H Yoshikawa M Sugiura N Kondo F. Accumulation of Copper in the Liver and Hepatic Injury in Chronic Hepatitis C. J Gastroenterol Hepatol. (2000) 15:786–91. doi: 10.1046/j.1440-1746.2000.02199.x, PMID: 10937686

[132] Llovet JM Kelley RK Villanueva A Singal AG Pikarsky E Roayaie S. Hepatocellular carcinoma. Nat Rev Dis Primers. (2021) 7:7. doi: 10.1038/s41572-021-00245-6, PMID: 33479224 PMC13537433

[133] Nagaraju GP Dariya B Kasa P Peela S El-Rayes BF. Epigenetics in hepatocellular carcinoma. Semin Cancer Biol. (2022) 86:622–32. doi: 10.1016/j.semcancer.2021.07.017, PMID: 34324953

[134] Guan D Zhao L Shi X Ma X Chen Z. Copper in cancer: from pathogenesis to therapy. Biomedicine pharmacotherapy = Biomedecine pharmacotherapie. (2023) 163:114791. doi: 10.1016/j.biopha.2023.114791, PMID: 37105071

[135] Ramchandani D Berisa M Tavarez DA Li Z Miele M Bai Y. Copper depletion modulates mitochondrial oxidative phosphorylation to impair triple negative breast cancer metastasis. Nat Commun. (2021) 12:7311. doi: 10.1038/s41467-021-27559-z, PMID: 34911956 PMC8674260

[136] Zhang X Yang Q. Association between serum copper levels and lung cancer risk: A meta-analysis. J Int Med Res. (2018) 46:4863–73. doi: 10.1177/0300060518798507, PMID: 30296873 PMC6300955

[137] Ge EJ Bush AI Casini A Cobine PA Cross JR DeNicola GM. Connecting copper and cancer: from transition metal signalling to metalloplasia. Nat Rev Cancer. (2022) 22:102–13. doi: 10.1038/s41568-021-00417-2, PMID: 34764459 PMC8810673

[138] Tamai Y Iwasa M Eguchi A Shigefuku R Sugimoto K Hasegawa H. Serum copper, zinc and metallothionein serve as potential biomarkers for hepatocellular carcinoma. PloS One. (2020) 15:e0237370. doi: 10.1371/journal.pone.0237370, PMID: 32857769 PMC7455040

[139] Li X Wang J Guo Z Ma Y Xu D Fan D. Copper metabolism-related risk score identifies hepatocellular carcinoma subtypes and SLC27A5 as a potential regulator of cuproptosis. Aging. (2023) 15:15084–113. doi: 10.18632/aging.205334, PMID: 38157255 PMC10781498

[140] Pang Z. Copper metabolism in hepatocellular carcinoma: from molecular mechanisms to therapeutic opportunities. Front Mol Biosci. (2025) 12:1578693. doi: 10.3389/fmolb.2025.1578693, PMID: 40433591 PMC12106024

[141] Liu X Sun B Yao Y Lai L Wang X Xiong J. Identification of copper metabolism and cuproptosis-related subtypes for predicting prognosis tumor microenvironment and drug candidates in hepatocellular carcinoma. Front Immunol. (2022) 13:996308. doi: 10.3389/fimmu.2022.996308, PMID: 36275743 PMC9582144

[142] Davis CI Gu X Kiefer RM Ralle M Gade TP Brady DC. Altered copper homeostasis underlies sensitivity of hepatocellular carcinoma to copper chelation. Metallomics: integrated biometal Sci. (2020) 12:1995–2008. doi: 10.1039/d0mt00156b, PMID: 33146201 PMC8315290

[143] Das SK Lewis BA Levens D. MYC: A complex problem. Trends Cell Biol. (2023) 33:235–46. doi: 10.1016/j.tcb.2022.07.006, PMID: 35963793 PMC9911561

[144] Sequera C Grattarola M Holczbauer A Dono R Pizzimenti S Barrera G. MYC and MET cooperatively drive hepatocellular carcinoma with distinct molecular traits and vulnerabilities. Cell Death Dis. (2022) 13:994. doi: 10.1038/s41419-022-05411-6, PMID: 36433941 PMC9700715

[145] Porcu C Antonucci L Barbaro B Illi B Nasi S Martini M. Copper/MYC/CTR1 interplay: A dangerous relationship in hepatocellular carcinoma. Oncotarget. (2018) 9:9325–43. doi: 10.18632/oncotarget.24282, PMID: 29507693 PMC5823635

[146] Elchuri S Oberley TD Qi W Eisenstein RS Jackson Roberts L Van Remmen H. CuZnSOD deficiency leads to persistent and widespread oxidative damage and hepatocarcinogenesis later in life. Oncogene. (2005) 24:367–80. doi: 10.1038/sj.onc.1208207, PMID: 15531919

[147] Das A Ash D Fouda AY Sudhahar V Kim YM Hou Y. Cysteine oxidation of copper transporter CTR1 drives VEGFR2 signalling and angiogenesis. Nat Cell Biol. (2022) 24:35–50. doi: 10.1038/s41556-021-00822-7, PMID: 35027734 PMC8851982

[148] Yee EMH Brandl MB Pasquier E Cirillo G Kimpton K Kavallaris M. Dextran-catechin inhibits angiogenesis by disrupting copper homeostasis in endothelial cells. Sci Rep. (2017) 7:7638. doi: 10.1038/s41598-017-07452-w, PMID: 28794411 PMC5550437

[149] Hancock JL Kalimutho M Straube J Lim M Gresshoff I Saunus JM. COMMD3 loss drives invasive breast cancer growth by modulating copper homeostasis. J Exp Clin Cancer research: CR. (2023) 42:90. doi: 10.1186/s13046-023-02663-8, PMID: 37072858 PMC10111822

[150] Cheng W Cheng Z Zhang C Weng L Xing D Zhang M. Investigating the association between commd3 expression and the prognosis of hepatocellular carcinoma. J Cancer. (2022) 13:1871–81. doi: 10.7150/jca.62454, PMID: 35399735 PMC8990410

[151] Zhu T Peng X Cheng Z Gong X Xing D Cheng W. COMMD3 expression affects angiogenesis through the hif1α/VEGF/NF-κB signaling pathway in hepatocellular carcinoma *in vitro* and *in vivo* . Oxid Med Cell Longevity. (2022) 2022:1655502. doi: 10.1155/2022/1655502, PMID: 36092163 PMC9463002

[152] Yoshii J Yoshiji H Kuriyama S Ikenaka Y Noguchi R Okuda H. The copper-chelating agent, trientine, suppresses tumor development and angiogenesis in the murine hepatocellular carcinoma cells. Int J Cancer. (2001) 94:768–73. doi: 10.1002/ijc.1537, PMID: 11745476

[153] Yoshiji H Kuriyama S Yoshii J Ikenaka Y Noguchi R Yanase K. The copper-chelating agent, trientine, attenuates liver enzyme-altered preneoplastic lesions in rats by angiogenesis suppression. Oncol Rep. (2003) 10:1369–73. doi: 10.3892/or.10.5.1369, PMID: 12883709

[154] Moriguchi M Nakajima T Kimura H Watanabe T Takashima H Mitsumoto Y. The Copper Chelator Trientine Has an Antiangiogenic Effect against Hepatocellular Carcinoma, Possibly through Inhibition of Interleukin-8 Production. Int J Cancer. (2002) 102:445–52. doi: 10.1002/ijc.10740, PMID: 12432545

[155] Ding P Zhang X Jin S Duan B Chu P Zhang Y. Cd147 functions as the signaling receptor for extracellular divalent copper in hepatocellular carcinoma cells. Oncotarget. (2017) 8:51151–63. doi: 10.18632/oncotarget.17712, PMID: 28881637 PMC5584238

[156] Huang Q Li J Xing J Li W Li H Ke X. CD147 promotes reprogramming of glucose metabolism and cell proliferation in HCC cells by inhibiting the P53-dependent signaling pathway. J Hepatol. (2014) 61:859–66. doi: 10.1016/j.jhep.2014.04.035, PMID: 24801417

[157] Niu D Wang D Fan L Liu Z Chen M Zhang W. The copper (II) complex of salicylate phenanthroline inhibits proliferation and induces apoptosis of hepatocellular carcinoma cells. Environ Toxicol. (2023) 38:1384–94. doi: 10.1002/tox.23771, PMID: 36891644

[158] Jiang M Yan Q Fu Y Meng L Gai S Pan X. Development of Cu(II) 4-hydroxybenzoylhydrazone complexes that induce mitochondrial DNA damage and mitochondria-mediated apoptosis in liver cancer. J inorganic Biochem. (2024) 256:112550. doi: 10.1016/j.jinorgbio.2024.112550, PMID: 38599004

[159] Shi X Shi D Yin Y Wu Y Chen W Yu Y. Cuproptosis-associated genes (CAGs) contribute to the prognosis prediction and potential therapeutic targets in hepatocellular carcinoma. Cell signalling. (2024) 117:111072. doi: 10.1016/j.cellsig.2024.111072, PMID: 38307306

[160] Li S Weng J Xiao C Lu J Cao W Song F. Cuproptosis-related molecular patterns and gene (ATP7a) in hepatocellular carcinoma and their relationships with tumor immune microenvironment and clinical features. Cancer Rep (Hoboken NJ). (2023) 6:e1904. doi: 10.1002/cnr2.1904, PMID: 37885090 PMC10728522

[161] Chen Y Tang L Huang W Abisola FH Zhang Y Zhang G. Identification of a prognostic cuproptosis-related signature in hepatocellular carcinoma. Biol direct. (2023) 18:4. doi: 10.1186/s13062-023-00358-w, PMID: 36750831 PMC9903524

[162] Quan Y Li W Yan R Cheng J Xu H Chen L. Tumor cuproptosis and immune infiltration improve survival of patients with hepatocellular carcinoma with a high expression of ferredoxin 1. Front Oncol. (2023) 13:1168769. doi: 10.3389/fonc.2023.1168769, PMID: 37361595 PMC10285401

[163] Quan B Liu W Yao F Li M Tang B Li J. LINC02362/hsa-miR-18a-5p/FDX1 axis suppresses proliferation and drives cuproptosis and oxaliplatin sensitivity of hepatocellular carcinoma. Am J Cancer Res. (2023) 13:5590–609., PMID: 38058825 PMC10695789

[164] Wu C Long L Wang M Shen L Hu J Tang H. Copper-mediated SEC14L3 promotes cuproptosis to inhibit hepatocellular carcinoma growth via ERK/YY1/FDX1 axis. Commun Biol. (2025) 8:658. doi: 10.1038/s42003-025-08101-z, PMID: 40274982 PMC12022014

[165] Sun B Ding P Song Y Zhou J Chen X Peng C. FDX1 downregulation activates mitophagy and the PI3K/AKT signaling pathway to promote hepatocellular carcinoma progression by inducing ROS production. Redox Biol. (2024) 75:103302. doi: 10.1016/j.redox.2024.103302, PMID: 39128228 PMC11366913

[166] Xia C Chen Y Zhu Y Chen D Sun H Shen T. Identification of DLAT as a potential therapeutic target via a novel cuproptosis-related gene signature for the prediction of liver cancer prognosis. J gastrointestinal Oncol. (2024) 15:2230–51. doi: 10.21037/jgo-24-609, PMID: 39554575 PMC11565118

[167] Li Z Zhou H Zhai X Gao L Yang M An B. MELK promotes HCC carcinogenesis through modulating cuproptosis-related gene DLAT-mediated mitochondrial function. Cell Death Dis. (2023) 14:733. doi: 10.1038/s41419-023-06264-3, PMID: 37949877 PMC10638394

[168] Li X Tang C Ye H Fang C. Injectable hydrogel-encapsulating pickering emulsion for overcoming lenvatinib-resistant hepatocellular carcinoma via cuproptosis induction and stemness inhibition. Polymers. (2024) 16:2418. doi: 10.3390/polym16172418, PMID: 39274051 PMC11397159

[169] Meng X Peng X Ouyang W Li H Na R Zhou W. Musashi-2 deficiency triggers colorectal cancer ferroptosis by downregulating the MAPK signaling cascade to inhibit HSPB1 phosphorylation. Biol procedures Online. (2023) 25:32. doi: 10.1186/s12575-023-00222-1, PMID: 38041016 PMC10691036

[170] Guo K Wang T Yin J Yang S Cui H Cao Z. Identification of cuproptosis-related patterns predict prognosis and immunotherapy response in hepatocellular carcinoma. J Cell Mol Med. (2024) 28:e70224. doi: 10.1111/jcmm.70224, PMID: 39663596 PMC11634814

[171] Mo JQ Zhang SY Li Q Chen MX Zheng YQ Xie X. Immunomodulation of cuproptosis and ferroptosis in liver cancer. Cancer Cell Int. (2024) 24:22. doi: 10.1186/s12935-023-03207-y, PMID: 38200525 PMC10777659

[172] Chen H Han Z Wang Y Su J Lin Y Cheng X. Targeting ferroptosis in bone-related diseases: facts and perspectives. J Inflammation Res. (2023) 16:4661–77. doi: 10.2147/jir.S432111, PMID: 37872954 PMC10590556

[173] Ren X Li Y Zhou Y Hu W Yang C Jing Q. Overcoming the compensatory elevation of NRF2 renders hepatocellular carcinoma cells more vulnerable to disulfiram/copper-induced ferroptosis. Redox Biol. (2021) 46:102122. doi: 10.1016/j.redox.2021.102122, PMID: 34482117 PMC8416961

[174] Zhang P Zhou C Ren X Jing Q Gao Y Yang C. Inhibiting the compensatory elevation of xCT collaborates with disulfiram/copper-induced GSH consumption for cascade ferroptosis and cuproptosis. Redox Biol. (2024) 69:103007. doi: 10.1016/j.redox.2023.103007, PMID: 38150993 PMC10788306

[175] Wang W Lu K Jiang X Wei Q Zhu L Wang X. Ferroptosis inducers enhanced cuproptosis induced by copper ionophores in primary liver cancer. J Exp Clin Cancer research: CR. (2023) 42:142. doi: 10.1186/s13046-023-02720-2, PMID: 37277863 PMC10242978

[176] Mao Z Nie Y Jia W Wang Y Li J Zhang T. Revealing prognostic and immunotherapy-sensitive characteristics of a novel cuproptosis-related LncRNA model in hepatocellular carcinoma patients by genomic analysis. Cancers. (2023) 15:544. doi: 10.3390/cancers15020544, PMID: 36672493 PMC9857215

[177] Zhou B Guo L Zhang B Liu S Zhang K Yan J. Disulfiram combined with copper induces immunosuppression via PD-L1 stabilization in hepatocellular carcinoma. Am J Cancer Res. (2019) 9:2442–55., PMID: 31815045 PMC6895448

[178] Yi M Niu M Xu L Luo S Wu K. Regulation of PD-L1 expression in the tumor microenvironment. J Hematol Oncol. (2021) 14:10. doi: 10.1186/s13045-020-01027-5, PMID: 33413496 PMC7792099

[179] Gu D Sun Y Wang J Sun J Lou H Kang W. Metformin regulates ferroptosis in skin cutaneous melanoma via ATF3/NRF2 axis. Cancer Genet. (2025) 294-295:136–44. doi: 10.1016/j.cancergen.2025.04.006, PMID: 40318300

[180] Liu H Tang T. Pan-cancer genetic analysis of disulfidptosis-related gene set. Cancer Genet. (2023) 278-279:91–103. doi: 10.1016/j.cancergen.2023.10.001, PMID: 37879141

[181] Wang R Lv Y Ni Z Feng W Fan P Wang Y. Intermittent hypoxia exacerbates metabolic dysfunction-associated fatty liver disease by aggravating hepatic copper deficiency-induced ferroptosis. FASEB journal: Off Publ Fed Am Societies Exp Biol. (2024) 38:e23788. doi: 10.1096/fj.202400840R, PMID: 38963329

